# N-Myc and STAT Interactor is an Endometriosis Suppressor

**DOI:** 10.3390/ijms25158145

**Published:** 2024-07-26

**Authors:** Yuri Park, Xiaoming Guan, Sang Jun Han

**Affiliations:** 1 Department of Molecular and Cellular Biology, Baylor College of Medicine, Houston, TX 77030, USA; yuri.park@bcm.edu; 2 Department of Obstetrics and Gynecology, Baylor College of Medicine, Houston, TX 77030, USA; xiaoming@bcm.edu; 3 Nuclear Receptor, Transcription and Chromatin Biology Program, Dan L. Duncan Cancer Center, Baylor College of Medicine, Houston, TX 77030, USA

**Keywords:** ERβ, NMI, HDAC8, apoptosis, necroptosis

## Abstract

In patients with endometriosis, refluxed endometrial fragments evade host immunosurveillance, developing into endometriotic lesions. However, the mechanisms underlying this evasion have not been fully elucidated. N-Myc and STAT Interactor (NMI) have been identified as key players in host immunosurveillance, including interferon (IFN)-induced cell death signaling pathways. NMI levels are markedly reduced in the stromal cells of human endometriotic lesions due to modulation by the Estrogen Receptor beta/Histone Deacetylase 8 axis. Knocking down NMI in immortalized human endometrial stromal cells (IHESCs) led to elevated RNA levels of genes involved in cell-to-cell adhesion and extracellular matrix signaling following IFNA treatment. Furthermore, NMI knockdown inhibited IFN-regulated canonical signaling pathways, such as apoptosis mediated by Interferon Stimulated Gene Factor 3 and necroptosis upon IFNA treatment. In contrast, NMI knockdown with IFNA treatment activated non-canonical IFN-regulated signaling pathways that promote proliferation, including β-Catenin and AKT signaling. Moreover, NMI knockdown in IHESCs stimulated ectopic lesions’ growth in mouse endometriosis models. Therefore, NMI is a novel endometriosis suppressor, enhancing apoptosis and inhibiting proliferation and cell adhesion of endometrial cells upon IFN exposure.

## 1. Introduction

Endometriosis is a medical condition in which endometrial tissues grow outside the uterus, such as on the ovaries and in the peritoneal cavity [1]. Approximately 10% of reproductive-aged women suffer from endometriosis. Symptoms include pelvic or abdominal pain, excessive menstrual flow, pain during urination or bowel movements, and infertility [1]. Conservative surgery is the first-line treatment for endometriosis; however, it cannot prevent a relapse of the condition [2]. Due to the estrogen dependence of endometriosis, anti-estrogen drugs have been used to alleviate symptoms, suppress post-operative recurrence, and enhance the quality of life for those affected [3,4]. However, hormonal therapy can lead to adverse effects, including post-menopausal symptoms and unintended consequences in other estrogen-responsive organs, such as the bones and brain [2,5]. Thus, new therapeutic approaches for endometriosis based on the discovery of a new molecular etiology are highly needed.

What causes endometriosis? Several hypotheses attempt to answer this question [6]. Among these, the retrograde menstruation hypothesis is a widely accepted explanation for the progression of endometriosis [1,7]. However, while 90% of reproductive-aged women experience retrograde menstruation, only 10% of them are diagnosed with endometriosis [8]. Therefore, other risk factors are also involved in initiating and progressing endometriosis along with retrograde menstruation. Previous studies have proposed that aberrant Estrogen Receptor beta (ERβ) expression in endometrial tissue might be a key driver in the development of endometriosis among reproductive-aged women who have experienced retrograde menstruation [9,10,11]. Hypomethylation of the ERβ promoter leads to the upregulation of ERβ in endometriotic lesions [12]. ERβ promotes the proliferation of endometriotic stromal cells by transcriptionally upregulating the Ras-like and estrogen-regulated growth inhibitor (RERG) in conjunction with prostaglandin [13]. Additionally, ERβ interacts with Tumor necrosis factor alpha (TNFα)-induced apoptosis complexes and inflammasomes, promoting endometriotic cell proliferation, adhesion, and invasion [14]. Interestingly, ERβ significantly suppresses the Interferon (IFN)-mediated cell death pathways, promoting the growth of endometriotic lesions in mice with endometriosis [15]. The aberrant expression of ERβ, in conjunction with retrograde menstruation, might contribute to the onset of endometriosis. However, the key question of how ERβ downregulates IFN-mediated cell death pathways is not addressed.

During retrograde menstruation, refluxed endometrial fragments activate host immune surveillance systems and elevate proinflammatory cytokines (such as TNFα and IFNs) in the pelvic area [16]. IFNA induces cell death in malignant cells through various mechanisms [17]. For example, IFNA activates the caspase-3-mediated intrinsic apoptotic pathway as well as caspase-4-mediated endoplasmic reticulum (ER) stress-induced apoptosis in HeLa cervical cancer cells [18]. In hepatocellular carcinoma cells, IFNA treatment enhances the expression of promyelocytic leukemia (PML) and tumor necrosis factor-related apoptosis-inducing ligand (TRAIL), activating the extrinsic apoptotic pathways that lead to cell death [19]. In the canonical IFNA pathway, Janus Kinase 1 (JAK1) and Tyrosine Kinase 2 (TYK2) phosphorylate Signal Transducers and Activators of Transcription 1 (STAT1) and 2 (STAT2). These phosphorylated forms subsequently dimerize and, in collaboration with Interferon Regulatory Factor 9 (IRF9), bind to the Interferon-Stimulated Response Element (ISRE) [20]. In endometriosis, type I IFN (including IFNA and IFNB) is dysregulated [16,21]. The mRNA levels of IFNα and its receptor 2 (IFNAR2) are elevated in the eutopic endometrium of endometriosis patients compared to healthy women [22]. A key mediator of the type I IFN pathway, JAK1, is significantly upregulated in endometriotic lesions compared to the eutopic endometrium of endometriosis patients. This suggests that the IFNA/IFNAR2/JAK1 signaling pathway is essential in endometriosis progression [16,23]. However, the transition from IFNA-mediated cell death signaling to IFNA-mediated growth stimulation signaling in endometriotic lesions, as opposed to the normal endometrium, remains unclear in the context of endometriosis progression.

Our microarray analysis demonstrated a significant reduction in N-Myc and STAT Interactor (NMI) levels within endometriotic lesions, compared to the normal endometrium of mice with endometriosis [15]. *NMI* is an interferon-inducible gene, with its promoter region containing an ISRE [24,25]. NMI interacts with all STATs except STAT2 [26]. Although NMI does not possess an intrinsic transcriptional activation domain, it enhances IL-2 and IFNG signaling. This enhancement occurs through the facilitation of STAT5 and STAT1 association with the transcriptional coactivators CBP/p300 [26]. NMI exhibits anti-tumor activity, as evidenced by its significantly low expression levels in breast cancer cells. Furthermore, the downregulation of NMI is associated with the promotion of human lung adenocarcinoma and low expression levels of NMI correlate with a poor prognosis in lung cancer [27,28]. In glioblastoma, however, NMI exhibits oncogenic activity, as its expression is positively correlated with poor prognosis. This is because NMI promotes cell cycle progression and proliferation through STAT1 [29].

Although the precise role of NMI in the progression of endometriosis remains unclear, our study has identified NMI’s function within this context: NMI acts as a suppressor of endometriosis. The downregulation of NMI, mediated by the Estrogen Receptor beta/Histone Deacetylase 8 axis, triggers key endometriotic phenotypes—such as anti-apoptosis, hyperproliferation, and increased cell adhesion—in endometrial cells, leading to the development of endometriotic lesions.

## 2. Results

### 2.1. NMI Levels Are Reduced in Stromal Cells of Human Endometriotic Lesions

**Figure 1:**
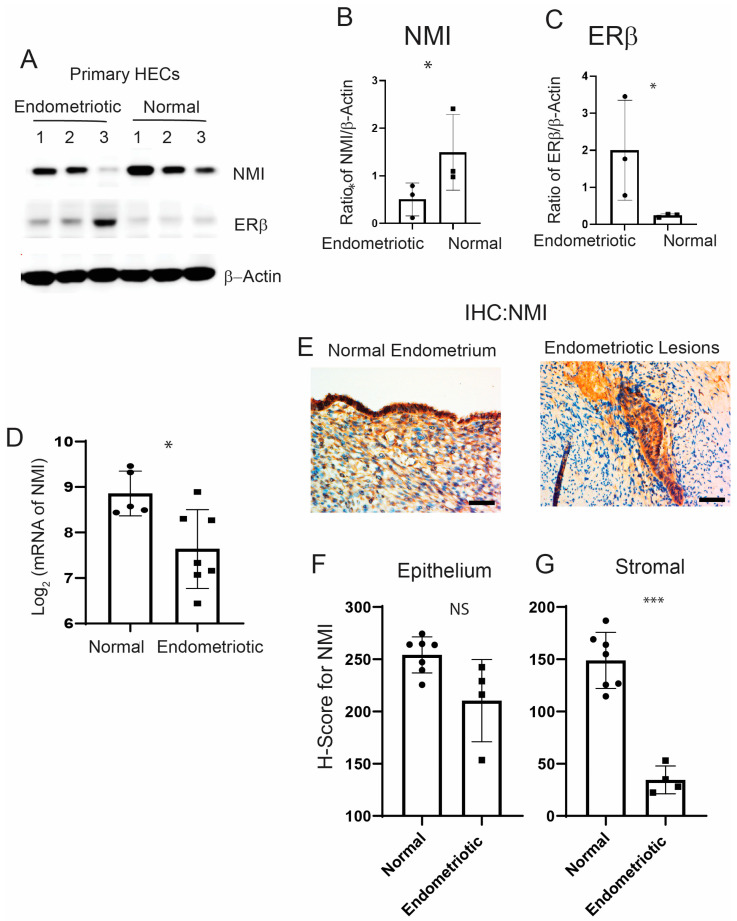
Reduced levels of NMI in endometriotic Lesions. (**A**) Protein levels of NMI, ERβ, and β-Actin in primary human endometrial stromal cells isolated from patients with endometrioma endometriosis (endometriosis) compared to those from normal endometrium (normal), as determined by Western blot analysis. (**B**) The protein ratio of NMI to β-Actin is shown in panel (**A**). (**C**) The protein ratio of ERβ to β-Actin is shown in panel (**A**). (**D**) Comparative analysis of NMI mRNA levels in human ectopic lesions versus normal endometrium, based on GSE25628 data. (**E**) Immunohistochemical analysis of NMI expression in normal human endometrium and human endometriotic lesions. Scale bar represents 100 µm. (**F**) Quantification of NMI protein levels in the epithelial compartment of both the endometrium and endometriotic lesions, as shown in panel (**E**). (**G**) Quantification of NMI protein levels in the stromal compartment of both the endometrium and endometriotic lesions, as illustrated in panel (**E**). NS: Non-Specific. Significance levels are indicated as follows: * *p* < 0.05 and *** *p* < 0.001, NS: Non-Specific determined by Student’s *t*-test.

We utilized primary human endometrial stromal cells derived from ectopic lesions in patients with endometriosis and from normal endometrium that we have isolated [30]. We subsequently measured NMI levels in both types of endometrial cells using Western blot analysis. NMI levels were found to be lower in human endometrial stromal cells from endometriosis patients compared to those in normal endometrial stromal cells (Figure 1A,B). To confirm that these endometrial stromal cells originated from endometriosis patients, we assessed ERβ levels in the cells, given that ERβ levels are known to be elevated in endometriotic lesions compared to normal endometrium [14]. In line with these observations, ERβ levels were significantly higher in stromal cells from endometriosis patients compared to normal endometrial stromal cells (Figure 1A,C). Furthermore, NMI expression was found to be inversely correlated with ERβ levels when comparing human endometriotic stromal cells with normal endometrial stromal cells. Next, we evaluated NMI levels in ectopic lesions from human endometriosis patients compared to normal endometrium using the GSE25628 dataset from the Gene Expression Omnibus (GEO) database, which is specific to endometriosis patients (Figure 1D). We found that NMI mRNA levels in human ectopic lesions were significantly lower than those in the normal endometrium. Furthermore, we examined NMI protein levels in human ectopic lesions relative to normal endometrium using immunohistochemistry (IHC). The H-Score for NMI indicated that NMI protein levels in the epithelium of ectopic lesions were like those in the normal endometrial epithelium (Figure 1E,F). However, NMI protein levels in the stromal cells of ectopic lesions were significantly lower than those in normal endometrium (Figure 1G). This significant reduction in stromal NMI levels within endometriotic lesions, compared to normal endometrium, suggests that the decline in stromal NMI may be associated with the progression of endometriosis.

### 2.2. The ERβ/HDAC8 Axis Leads to the Reduction in NMI Expression in Endometrial Stromal Cells

**Figure 2:**
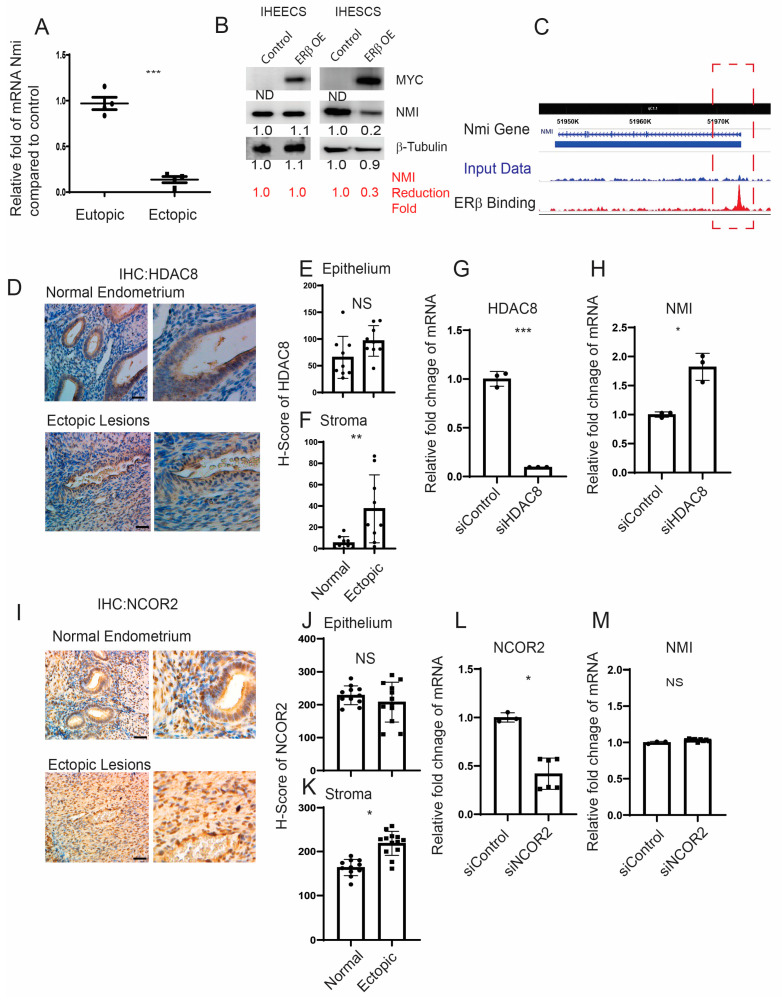
Modulation of NMI expression via the ERβ/HDAC8 axis in endometrial stromal cells. (**A**) Comparative analysis of NMI mRNA levels in ERβ-overexpressing eutopic endometrium and ectopic lesions versus control samples, illustrating the relative fold changes in NMI RNA. (**B**) Evaluation of NMI protein levels in ERβ-overexpressing immortalized human endometrial epithelial cells (IHEECs) and stromal cells (IHESCs) via Western blot. β-Tubulin served as a normalization control for NMI expression levels, while MYC-tagged ERβ levels were assessed using a MYC antibody. (**C**) ChIP-seq analysis in ERβ-overexpressing mouse ectopic lesions highlighted ERβ’s binding to the NMI locus promoter region as marked in the red dotted box, indicating direct regulatory actions. (**D**) HDAC8 protein expression in normal endometrium and ovarian endometriotic lesions was analyzed through immunohistochemistry using an HDAC8-specific antibody. Scale bar represents 100 µm. (**E**,**F**) Quantitative assessment of HDAC8 protein concentrations within the epithelial (**E**) and stromal (**F**) compartments of normal endometrium and ovarian endometriotic lesions, as shown in panel (**D**). (**G**) Quantitative RT-PCR was employed to measure HDAC8 mRNA levels in IHESCs treated with either control siRNA (siControl) or HDAC8-targeting siRNA (siHDAC8). (**H**) NMI mRNA levels in IHESCs subjected to siControl or siHDAC8 treatments were quantified using quantitative RT-PCR. (**I**) NCOR2 protein expression in normal endometrium and ovarian endometriotic lesions was determined via immunohistochemistry, utilizing an NCOR2 antibody. Scale bar represents 100µm. (**J**,**K**) Quantitative analysis of NCOR2 protein levels in the epithelial (**J**) and stromal (**K**) compartments of normal endometrium and ovarian endometriotic lesions, corresponding to panel (**I**). (**L**) Quantitative RT-PCR was used to ascertain NCOR2 mRNA levels in IHESCs treated with siControl or NCOR2-specific siRNA (siNCOR2). (**M**) Assessment of NMI mRNA levels in IHESCs following treatment with siControl or siNCOR2, determined by quantitative RT-PCR. * *p* < 0.05, ** *p* < 0.01, and *** *p* < 0.001, NS: Non-Specific determined by Student’s *t*-test.

We generated ERβ-overexpressing endometriotic lesions and eutopic endometrium by auto-transplanting uterine fragments from an endometrium-specific ERβ-overexpressing female mouse back into the same mouse [14]. For the control group in our endometriosis study, endometriosis was induced by auto-transplanting uterine fragments from control female mice back into the same mouse. Endometriotic lesions and eutopic endometrium were then isolated from both endometrium-specific ERβ-overexpressing mice and their control counterparts with endometriosis. Compared to control ectopic lesions, NMI levels were significantly lower in ectopic lesions from ERβ-overexpressing mice, as evidenced by the NMI ratio in ERβ-overexpressing ectopic lesions to normal ectopic lesions being 0.2 (Figure 2A) [15].

However, no significant difference in NMI levels was observed between ERβ-overexpressing and wild-type (WT) control eutopic endometrium (Figure 2A). To further validate our findings, we employed Myc-tagged ERβ-overexpressing immortalized human endometrial epithelial cells (IHEECs) and immortalized human endometrial stromal cells (IHESCs) lines that we had previously developed and validated [14]. MYC-tagged ERβ was robustly expressed in both cell types, as confirmed through Western blot analysis using an MYC-antibody (Figure 2B). The overexpression of ERβ significantly decreased NMI expression in IHESCs but not in IHEECs when compared to their respective parental cells. This pattern aligns with the stromal cell-specific reduction in NMI levels observed in human endometriotic lesions (Figure 1E), indicating a reverse correlation between ERβ and NMI in endometriotic lesions. The subsequent inquiry focuses on how ERβ reduces NMI levels in endometriotic lesions. To unravel this crucial question, we investigated the potential interaction between ERβ and the promoter regions of NMI genomic loci within endometriotic lesions, utilizing our lesion-specific ERβ ChIP-seq dataset [15]. ERβ ChIP-seq analysis revealed direct binding of ERβ to the NMI promoter region within endometriotic lesions (Figure 2C). In both normal and endometriotic lesions, ERβ can bind to the promoter region of NMI. However, endometriotic lesions have higher levels of ERβ and local estrogen compared to the normal endometrium. The abundant ERβ actively binds to the NMI promoter region under high estrogen levels in endometriotic lesions, compared to normal endometrium. Therefore, ERβ-mediated repression of NMI is detected in endometriotic lesions but not in normal endometrium.

This raises the question: how does ERβ downregulate NMI levels in human endometrial stromal cells? Several corepressors have been identified that interact with ERβ, thereby inhibiting its activity [31,32]. Among them, Histone Deacetylase 8 (HDAC8) is aberrantly expressed in endometriosis compared to normal tissues [33]. Immunohistochemical (IHC) analysis comparing human endometriotic lesions with normal endometrium revealed that HDAC8 levels were increased in the stromal cells but not in the epithelial cells of human ectopic lesions relative to the normal endometrium (Figure 2D–F). To investigate the role of HDAC8 in modulating NMI expression in IHESCs, we utilized HDAC8 siRNA to generate HDAC8 knockdown IHESCs (Figure 2G). Non-target siRNA served as the control. HDAC8 siRNA significantly decreased HDAC8 RNA levels in IHESCs compared to the control siRNA (Figure 2G). Knocking down HDAC8 using HDAC8 siRNA resulted in an increased RNA level of NMI in IHESCs compared to cells treated with non-target siRNA (Figure 2H). This indicates that the elevation of HDAC8 in the stroma is associated with decreased NMI levels in endometriotic lesions. Similarly, the level of Nuclear receptor corepressor 2 (NCOR2) was higher in the stromal cells but not in the epithelial cells of human ectopic lesions (Figure 2I–K). To explore NCOR2’s influence on NMI expression, we reduced the RNA levels of NCOR2 in IHESCs using NCOR2 siRNA, with a control siRNA for comparison (Figure 2L). However, decreasing NCOR2 did not result in elevated NMI RNA levels in IHESCs compared to the control (Figure 2M). Thus, unlike HDAC8, the elevation of NCOR2 does not play a role in suppressing NMI levels in the stroma of endometriotic lesions. 

### 2.3. Reducing NMI Levels Induced an Anti-Apoptotic Phenotype in Human Endometrial Stromal Cells Following IFNA Treatment

**Figure 3:**
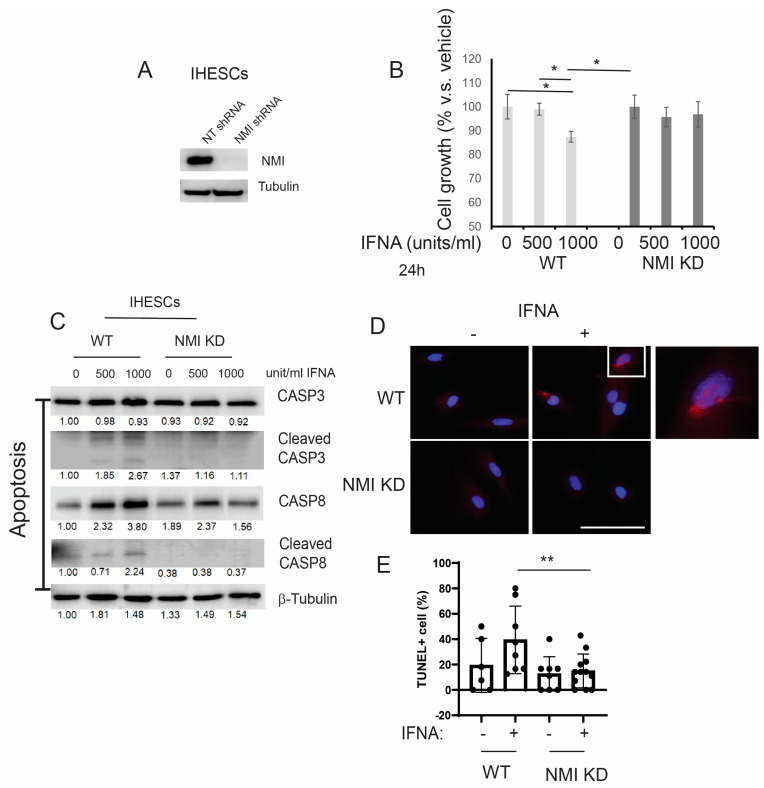
Suppression of IFNα-induced apoptosis in human endometrial stromal cells by NMI knockdown (KD). (**A**) Assessment of NMI protein levels in IHESCs treated with non-targeting shRNA (NT shRNA) and NMI-specific shRNA, analyzed via Western blot using an NMI antibody. β-Tubulin was employed as a normalization standard for NMI protein expression. (**B**) Evaluation of cell proliferation in control IHESCs and NMI KD-IHESCs following treatment with 0, 500, or 1000 units/mL of IFNA for 24 h, indicating the impact of NMI KD on cell growth under IFNA exposure. (**C**) Analysis of apoptosis signaling pathways in control KD (wild-type, WT) and NMI KD IHESCs post-treatment with 0, 500, or 1000 units/mL of IFNA for 24 h. Protein levels of caspase-3 (CASP3), cleaved CASP3, caspase-8 (CASP8), and cleaved CASP8 were quantified via Western blot. β-Tubulin levels served as a normalization control for the apoptosis-associated proteins. (**D**) Representative images of TUNEL assay in WT and NMI KD IHESCs post-treatment with 0 or 500 units/mL of IFNA for 24 h. TUNEL-positive cells and nuclei were stained in red and blue, respectively. The scale bar represents 100 µm. (**E**) Quantitative analysis of TUNEL positivity from (**D**). * *p <* 0.05 and ** *p* < 0.01 determined by one-side ANOVA with a post-hoc Tukey test.

IFNA levels are elevated in patients with endometriosis [16] and the elevated IFNA causes apoptosis in various cells, including cancer cells [17,19]. Compared to normal endometrium, a distinctive molecular phenotype of endometriotic cells is their resistance to apoptosis in response to cell death signaling, including IFNA [34]. To evaluate if the down-regulation of NMI imparts anti-apoptotic properties in human endometrial stromal cells, countering IFNA-induced cell death signaling, we generated stable NMI-knockdown (KD) IHESC lines (NMI KD-IHESCs) through the introduction of lentiviruses carrying an NMI shRNA expression unit. For control purposes, IHESCs were transduced with a lentivirus containing a non-targeting (NT) shRNA expression unit. Compared to the NT shRNA transduced cells, NMI protein levels were significantly reduced in the NMI KD-IHESCs (Figure 3A). Subsequently, we investigated the impact of NMI knockdown (KD) on IFNA-induced cell death in IHESCs. Treatment with IFNA significantly inhibited the growth of IHESCs compared to vehicle control (Figure 3B). In contrast, IFNA treatment did not suppress the growth of NMI KD-IHESCs (Figure 3B). Thus, NMI KD conferred resistance to apoptosis in IHESCs upon exposure to IFNA.

Our previous study demonstrated that ERβ inhibits IFNA-mediated apoptosis in human endometrial epithelial cells by interacting with the apoptosis machinery [15]. The levels of IFNA in endometriosis patient samples are still unknown, while the IFNA serum level is approximately 25 pg/mL (13.87 units/mL) in healthy individuals [35]. In IFNA adjuvant trials, doses are categorized as low-dose (<3 MU), intermediate-dose (5-10 MU), and high-dose (>10 MU). It is established that an in vitro concentration of 1000 units/mL corresponds to a dose of 20 MU/m^2^ in the adjuvant therapy of melanoma [36,37]. According to these criteria, the doses of IFNA used in our study are considered intermediate (500 units/mL) and high (1000 units/mL), which are more relevant in a therapeutic context than in a natural context. To determine if the loss of NMI also contributes to the prevention of IFNA-induced apoptosis, we exposed both IHESCs and NMI KD-IHESCs to 0, 500, and 1000 units/mL of human recombinant IFNA proteins and analyzed apoptosis signaling through Western blot analysis (Figure 3C). Treatment with IFNA heightened apoptosis signaling in IHESCs, as evidenced by increased levels of the cleaved forms of CASP3 and CASP8 following IFNA stimulation (Figure 3C). Conversely, in NMI KD-IHESCs, NMI knockdown thwarted IFNA-induced apoptosis, indicated by the absence of cleaved CASP3 and CASP8 after IFNA treatment (Figure 3C). In addition to Western blot, TUNEL assay also showed that NMI KD significantly inhibited TUNEL positivity upon IFNA treatment compared to control IHESCs (Figure 3D,E). Consequently, NMI KD curtailed the IFNA-induced apoptosis signaling in IHESCs.

### 2.4. NMI KD Augmented the Expression of Genes Involved in Extracellular Matrix Signaling and Cell Adhesion in Human Endometrial Stromal Cells

**Figure 4:**
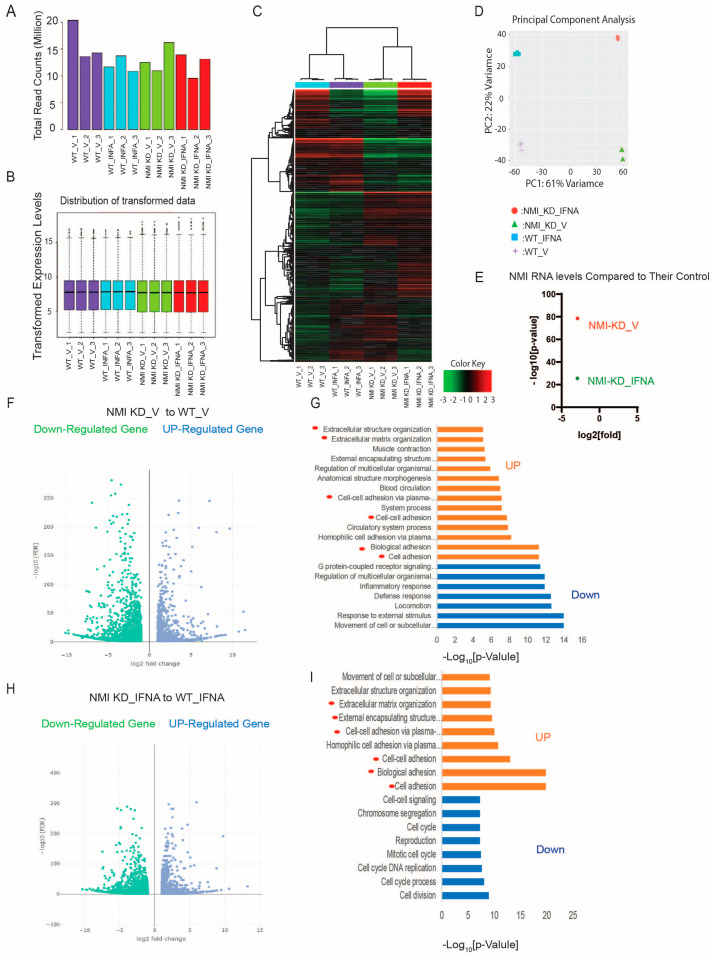
Enhancement of extracellular matrix and cell adhesion signaling in human endometrial stromal cells by NMI KD. (**A**) Total read counts of each library. (**B**) Boxplot representing transformed expression levels. (**C**) Hierarchical clustering and (**D**) PCA analysis depicting substantial differences in thousands of genes induced by NMI knockdown in IHESCs upon IFNA treatment. (**E**) Relative fold change in NMI RNA levels in NMI KD IHESCs as compared to control KD IHESCs upon vehicle (NMI-KD_V) and IFNA (NMI-KD_INFA) treatment. (**F**) Volcano plot to define the differential gene expression profile between NMI KD and control KD (WT) IHESCs under vehicle treatment. Identification of up- or down-regulated genes with >1 (−log10[FDR]) and >2 (log2[Fold]) changes in NMI KD IHESCs compared to WT control IHESCs under vehicle treatment. (**G**) Gene ontology analysis heightened the cellular pathways linked to the up-and down-regulated genes in NMI KD IHESCs as compared with control KD IHESCs in Panel (**F**). The cellular pathways related to extracellular matrix and cell-to-cell adhesion are highlighted with red circles. (**H**) Volcano plot showing differential gene expression profile between NMI KD and control KD (WT) control IHESCs under IFNA treatment (500 units/mL) for 24 h. Identification of up- or down-regulated genes with >1 (−log10[FDR]) and >2 (log2[Fold]) changes in NMI KD IHESCs compared to WT control IHESCs under IFNA treatment. (**I**) Gene ontology analysis heightened the cellular pathways linked to the up-and down-regulated genes in NMI KD IHESCs as compared with control KD IHESCs upon IFNA treatment in Panel (**H**). The cellular pathways related to extracellular matrix and cell-to-cell adhesion are highlighted with red circles.

To explore NMI’s involvement in endometriosis progression in depth, we examined the RNA expression profiles of NMI knockdown (NMI KD) in IHESC cells and their respective controls. These cells underwent treatment with either a vehicle or IFNA (1000 unit/mL) for a duration of 24 h, followed by RNA sequencing. The total read counts for each library, along with the distribution of the transformed data, were illustrated in a bar plot, revealing slight variations in library sizes (Figure 4A,B). The RNA sequencing process was meticulously performed for each sample. Through hierarchical clustering and Principal Component Analysis (PCA), we identified significant differences in the expression of thousands of genes affected by NMI knockdown in IHESCs. These differences were particularly pronounced when comparing the impact of vehicle and IFNA treatments (Figure 4C,D). Subsequently, we investigated the molecular phenotype alterations in IHESC cells due to NMI knockdown (NMI KD) under both the absence and presence of IFNA treatment. Initially, this investigation involved analyzing the RNA expression profiles of NMI KD IHESCs versus WT control IHESCs treated with the vehicle. This step aimed to elucidate NMI KD’s impact on IHESCs without IFNA induction. RNA-seq analysis revealed a substantial decrease in NMI RNA levels in NMI KD IHESCs compared to WT controls, with log2 fold changes in NMI RNA levels of −2.98 and −2.99 observed in the presence of both vehicle and IFNA, respectively (Figure 4E). This outcome confirmed the effective knockdown status of NMI in IHESCs used for RNA sequencing. We then utilized RNA-seq data to pinpoint genes significantly upregulated or downregulated in NMI KD IHESCs relative to WT control cells, adopting thresholds of >1 (−log10[FDR]) and > 2 (log2[Fold Change]). These findings are depicted in Figure 4F (Table S1). Further analysis employing Gene Ontology (GO) Biological Process terms highlighted a significant enhancement in extracellular matrix signaling and cell-to-cell adhesion signaling pathways in NMI KD IHESCs compared to WT controls, notably in the absence of IFNA stimulation (Figure 4G, Table S2).

Continuing from there, we assessed the modifications in the RNA expression profile of IHESCs following treatment with IFNA in the context of NMI knockdown. Once again, we identified genes exhibiting significant up- and down-regulation based on the same criteria, comparing NMI KD IHESCs to WT controls following IFNA treatment (Figure 4H, Table S3). Interestingly, the GO Biological Process analysis unveiled a parallel trend; extracellular matrix and cell-to-cell adhesion signaling pathways were notably enhanced in NMI KD IHSECs compared to WT controls, mirroring the outcomes observed following vehicle treatment (Figure 4I, Table S4).

**Figure 5:**
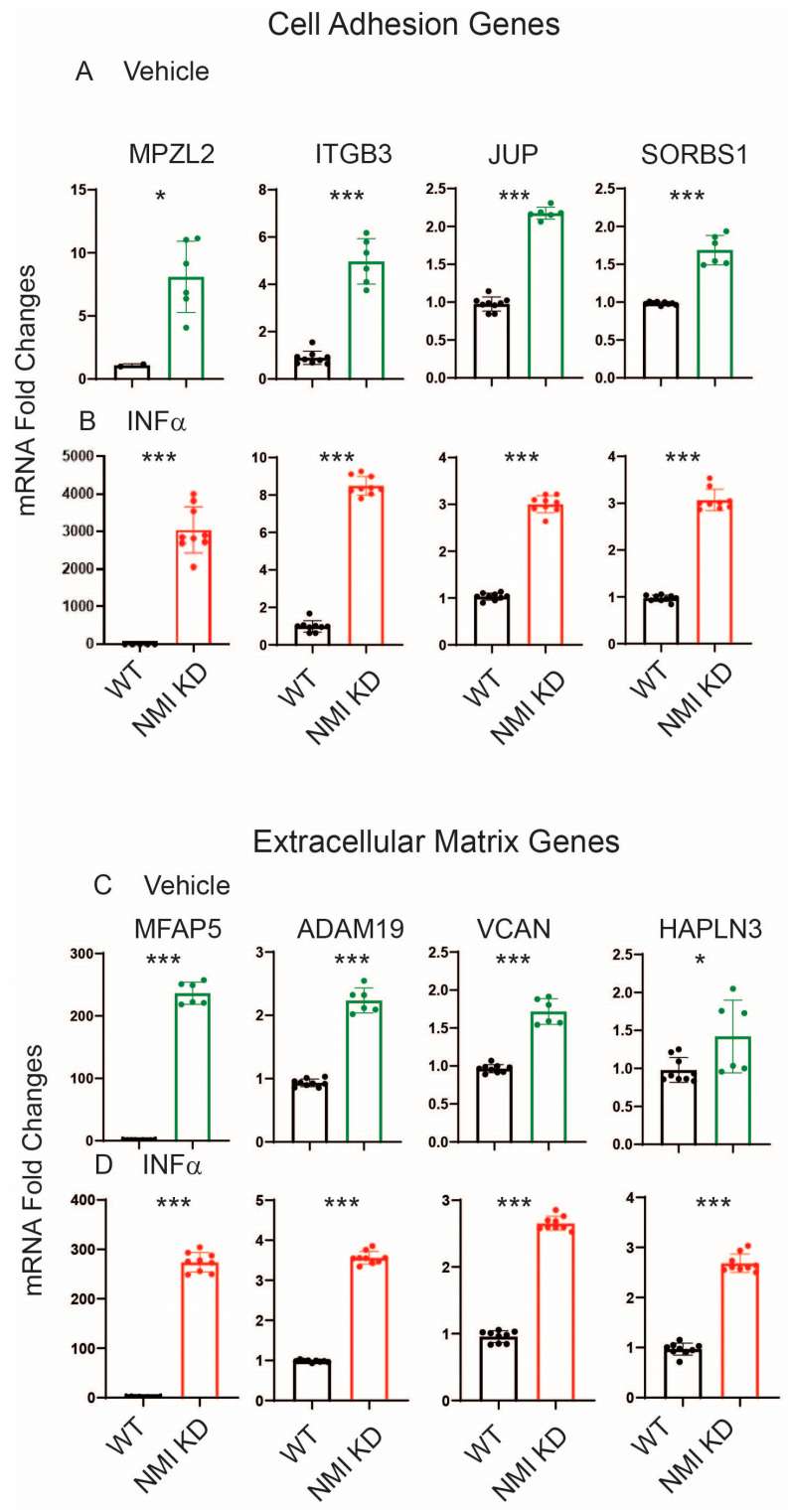
Upregulation of genes involved in cell adhesion and extracellular matrix signaling in NMI KD IHESCs compared to control KD IHESCs. (**A**) mRNA fold changes in cell adhesion-related genes (MPZL2, ITGB3, JUP, and SORBS1) in control KD (WT) versus NMI KD IHESCs with vehicle treatment and (**B**) IFNA treatment at 500 units/mL for 24 h. (**C**) mRNA fold changes in genes associated with extracellular matrix signaling (MFAP5, ADAM19, VCAN, and HAPLN3) in WT versus NMI KD IHESCs with vehicle treatment and (**D**) IFNA treatment at 500 units/mL for 24 h. Significance levels are indicated as follows: * *p* < 0.05 and *** *p* < 0.001 determined by Student’s *t*-test.

To validate RNA sequencing data, the genes MPZL2, ITGB3, JUP, and SORBS1, which are associated with cell adhesion, were chosen to investigate the impact of NMI KD on cell adhesion pathways in endometrial stromal cells. We assessed their mRNA levels in NMI KD and WT control IHESCs following treatment with either vehicle or IFNA using quantitative RT-PCR. NMI KD resulted in elevated mRNA levels of these genes in IHESCs under both conditions, with and without IFNA treatment, compared to the WT control (Figure 5A,B). Treatment with IFNA significantly increased the RNA levels of these genes in NMI KD-IHESCs in comparison to the vehicle treatment. Additionally, the mRNA levels of MFAP5, ADAM19, VCAN, and HAPLN3, which are implicated in extracellular matrix signaling, were evaluated in NMI KD and WT control IHESCs following treatment with vehicle or IFNA, using quantitative RT-PCR. NMI KD led to an increase in mRNA levels of all these genes involved in extracellular matrix signaling in IHESCs, in both the absence and presence of IFNA, when compared to the WT control (Figure 5C,D). Treatment with IFNA significantly enhanced the expression of these genes in NMI KD-IHESCs relative to the vehicle. In summary, the downregulation of NMI in IHESCs augmented signaling pathways associated with the extracellular matrix and cell adhesion, both crucial for the advancement of endometriosis in response to IFNA exposure.

### 2.5. NMI KD Inhibited the Apoptosis and Necroptosis Mediated by Interferon-Stimulated Genes (ISGs) in IHESCs upon Stimulation with IFNA, Affecting the IFNA-Regulated Canonical Pathway

Exposure to IFNA stimulates the formation of the Interferon-stimulated gene factor 3 (ISGF3) complex, composed of phosphorylated STAT1 (P-STAT1), phosphorylated STAT2 (P-STAT2), and Interferon Regulatory Factor 9 (IRF9); the ISGF3 complex binds to the Interferon-sensitive response element (ISRE) and then enhances the expression of ISGs, including those involved in cell death signaling [20,38]. Given the crucial role of the ISGF3 complex in IFNA signaling pathways [39], we postulated that knocking down NMI might hinder the formation of the IFNA-induced ISGF3 complex, thereby preventing IFNA-induced cell death signaling in endometriotic cells. 

**Figure 6:**
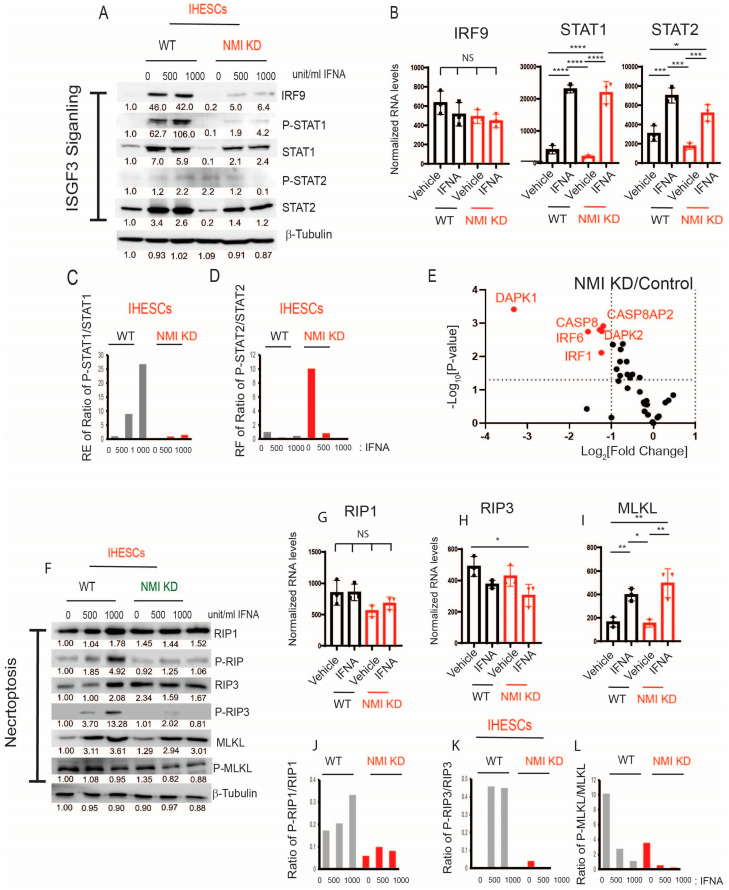
Suppression of ISGF3-mediated apoptosis and necroptosis in human endometrial stromal cells by NMI KD upon IFNA treatment. (**A**) Protein levels of ISGF3 complex components, IRF9, STAT1, p-STAT1(Tyr701), STAT2, and p-STAT2 (Tyr689) in control KD versus NMI KD IHESCs upon IFNA treatment determined by Western blotting. (**B**) RNA levels of IRF9, STAT1, and STAT3 normalized by 18sRNA in control KD versus NMI KD IHESCs upon IFNA treatment. (**C**) The ratio of p-STAT1/STAT1 and (**D**) the ratio of p-STAT2/STAT2 in control KD versus NMI KD IHESCs upon IFNA treatment determined by quantification of panel (**A**). (**E**) The relative fold change in RNA levels of genes involved in apoptosis in NMI KD IHESCs compared to control KD IHESCs upon IFNA treatment. (**F**) Protein levels of necroptosis components. RIP1, p-RIP1(Ser166), RIP3, p-RIP3(Ser227), MLKL, and p-MLKL(Ser358) in control KD (WT) versus NMI KD IHESCs following IFNA treatment. RNA levels of (**G**) RIP1, (**H**) IRIP3, and (**I**) MLKL normalized by 18sRNA in control KD versus NMI KD IHESCs upon IFNA treatment. The ratio of (**J**) p-RIP1/RIP1, (**K**) p-RIP2/RIP2, and (**L**) p-MLKL/MLKL in control KD versus NMI KD IHESCs upon IFNA treatment was determined by quantification of panel (**F**). Significance levels are indicated as follows: * *p* < 0.05, ** *p* < 0.01, *** *p* < 0.001, and **** *p* < 0.0001 determined by one-way AVONA with post-hoc Tukey test.

IFNA treatment led to a significant increase in the total protein expressions of IRF9, STAT1, phosphorylated STAT1 (p-STAT1), STAT2, and phosphorylated STAT2 (p-STAT2) in IHESCs, as opposed to vehicle-treated cells (Figure 6A). Contrary to protein levels, RNA-seq data indicated that NMI knockdown (NMI-KD) did not alter the RNA levels of IRF9 and STAT1/2 in IHESCs compared to the control (Figure 6B). Hence, IFNA treatment notably enhanced the formation of the ISGF3 complex in IHESCs. However, NMI knockdown resulted in reduced protein levels of IRF9, STAT1, and STAT2 in IHESCs upon IFNA stimulation, without a corresponding decrease in their RNA levels (Figure 6A,B). Additionally, NMI KD diminished the activation of STAT1 and STAT2, as determined by the ratios of p-STAT1/STAT1 and p-STAT2/STAT2, in IHESCs upon IFNA stimulation compared to control IHESCs (Figure 6C,D). Therefore, NMI may play a role in stabilizing the protein levels of IRF9, STAT1, and STAT2 upon IFNA stimulation, thus facilitating the formation of the ISGF3 complex and elevating ISGs. These genes are implicated in the progression of apoptosis by regulating apoptosis-associated genes [40]. RNA-seq analysis revealed that NMI knockdown (NMI KD) significantly decreased mRNA levels of apoptotic genes, including Death-Associated Protein Kinase 1 (DAPK1), DAPK2, Caspase 8 (CASP8), Caspase 8 Associated Protein 2 (CASP8AP2), Interferon Regulatory Factor 1 (IRF1), and IRF6 in IHESCs, in comparison to control IHESCs following IFNA stimulation (Figure 6E). Consequently, NMI KD markedly attenuated IFNA-induced apoptosis in IHESCs. This specific effect of NMI knockdown, which disrupts ISGF3 complex formation, contributes to the anti-apoptotic property of endometriotic lesions, countering cell death signaling such as IFNA-induced apoptosis.

Necroptosis, an alternative mode of cell death, is also regulated by IFN via the activation of Receptor-Interacting Protein Kinase 1 (RIP1) and Receptor-Interacting Protein Kinase 3 (RIP3) [41]. Type I IFN initiates the phosphorylation of RIP1 and RIP3, which subsequently recruits and activates Mixed Lineage Kinase Domain-Like (MLKL) to assemble the necrosome, thereby inducing cell death [41]. In macrophages, IFN induces necroptosis through ISGF3 signaling by persistently activating RIP3, ultimately leading to inflammation [39]. Given that NMI knockdown adversely affected ISGF3 signaling in endometrial stromal cells, we explored whether NMI knockdown could also inhibit necroptosis in these cells, potentially facilitating the progression of endometriosis. To assess necroptosis, we examined the protein levels of RIP1, RIP3, and MLKL in IHESCs. NMI knockdown did not affect the protein levels of these necroptosis markers in IHESCs following IFNA treatment (Figure 6F). Similarly, NMI knockdown did not alter the mRNA levels of RIP1, RIP3, and MLKL in IHESCs upon IFNA treatment (Figure 6G–I). Subsequently, we assessed the activation of RIP1, RIP3, and MLKL by examining their phosphorylated forms. 

We calculated the ratios of phosphorylated RIP1 (Ser166) (p-RIP1) to RIP1, phosphorylated RIP3 (Ser227) (p-RIP3) to RIP3, and phosphorylated MLKL (Ser358) (p-MLKL) to MLKL based on Western blot data (Figure 6F). IFNA treatment significantly increased the ratios of p-RIP1/RIP1 and p-RIP3/RIP3 in IHESCs compared to vehicle-treated cells (Figure 6J,K). However, NMI knockdown suppressed the elevation of the p-RIP1/RIP1 and p-RIP3/RIP3 ratios in IHESCs upon IFNA stimulation compared to the vehicle (Figure 6J,K), thereby preventing the activation of RIP1 and RIP3 in IHESCs upon IFNA stimulation. Interestingly, IFNA treatment decreased the ratio of p-MLKL/MLKL in IHESCs compared to vehicle-treated cells (Figure 6L). NMI knockdown significantly reduced the ratio of p-MLKL/MLKL in IHESCs upon IFNA stimulation compared to control IHESCs (Figure 6L). Therefore, NMI knockdown effectively suppressed the phosphorylation of necroptosis components upon IFNA treatment and hindered IFNA-induced necroptosis in endometrial stromal cells, potentially contributing to the progression of endometriosis.

### 2.6. NMI Knockdown Enhanced IFNA-Induced Activation of Non-Canonical Pathways in Human Endometrial Stromal Cells

**Figure 7:**
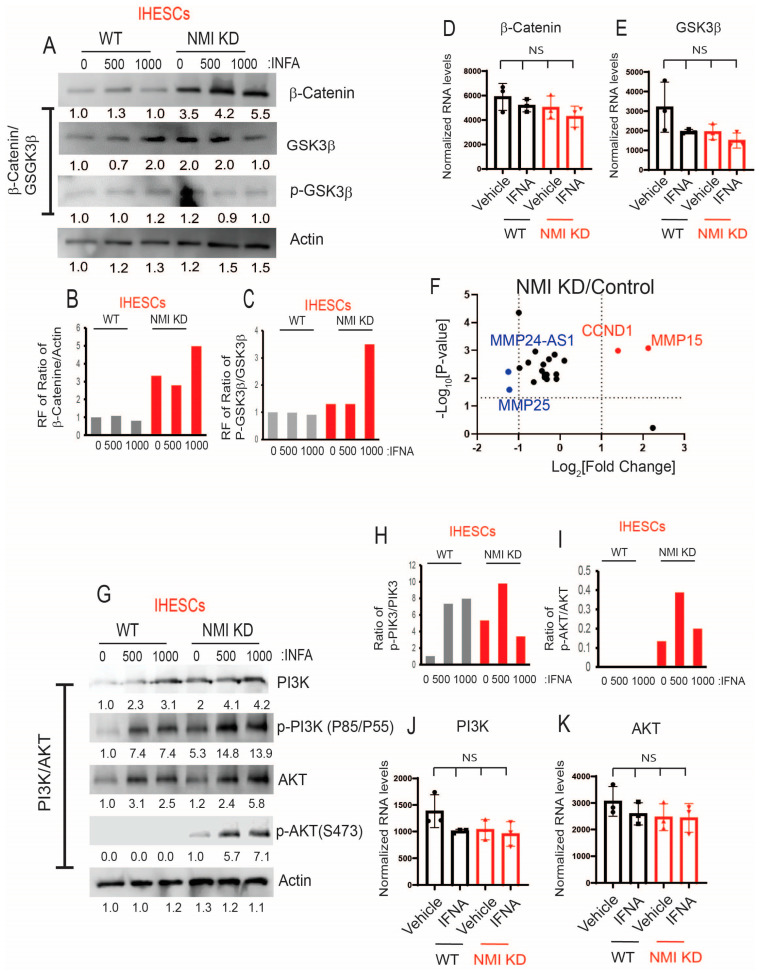
Increasing IFNA non-canonical pathways in endometrial stromal cells by NMI KD. (**A**) Protein levels of β-Catenin, GSK3β, and p-GSK3β (Ser9) in control KD (WT) versus NMI KD IHESCs upon IFNA treatment. Protein levels of b-actin were determined to normalize the β-Catenin/GSK3β axis. RNA levels of (**B**) β-Catenin and (**C**) GSK3β normalized by 18sRNA were determined in control KD (WT) and NMI KD IHESCS upon IFNA treatment. Relative fold change in (**D**) the ratio of β-Catenin/b-Actin and (**E**) the ratio of p-GSK3β/GSK3β in control KD (WT) and NMI KD IHESCS upon IFNA treatment were calculated based on panel A. (**F**) The relative fold change in RNA levels of β-Catenin target genes in NMI KD IHESCs compared to control KD IHESCs. (**G**) Protein levels of PI3K, p-PI3K (Tyr 85/Tyr 55), AKT, and p-AKT (Ser473) in control KD (WT) and NMI KD IHESCs upon IFNA treatment. Protein levels of b-actin were determined to normalize the PI3K/p-PI3K and the AKT/p-AKT axis. (**H**) Relative fold change in the ratio of p-PI3K(Tyr85/55)/PI3K in control KD (WT) and NMI KD IHESCs upon IFNA treatment was quantified based on panel G. (**I**) Relative fold change in the ratio of p-AKT(Ser473)/AKT in control KD (WT) and NMI KD IHESCS upon IFNA treatment were calculated based on panel G. (**J**) RNA levels of PI3K normalized by 18sRNA were determined in control KD (WT) and NMI KD IHESCS upon IFNA treatment. (**K**) RNA levels of AKT normalized by 18sRNA were determined in control KD (WT) and NMI KD IHESCS upon IFNA treatment. RF: Relative fold change, NS: Non-Specific. Significance levels are indicated as follows: NS, Non-Specific determined by one-side ANOVA with a post-hoc Tukey test.

While the JAK/STAT pathways are recognized as canonical pathways activated by IFNA, this cytokine also triggers various non-canonical pathways, including PI3K/AKT, GSK3β/β-catenin, and MAPK, to modulate cellular processes [20,42,43]. Furthermore, the activation of these non-canonical IFNA pathways plays a crucial role in the progression of endometriosis [44,45,46]. For example, the aberrant activation of Wnt/β-catenin signaling during the secretory phase of the menstrual cycle in endometriosis has been associated with excessive inactivation of GSK3β [45]. Furthermore, NMI inhibits AKT and GSK3β/β-catenin signaling in various cancers, thereby impeding cancer progression [27,28]. However, the biological functions of NMI in IFNA-induced non-canonical cellular pathways during the progression of endometriosis remain unexplored. To investigate this, we aimed to determine the effects of NMI knockdown on GSK3β/β-catenin and AKT signaling in endometrial stromal cells. Following IFNA treatment, there was no change in the protein levels of β-catenin but GSK3β levels were increased in IHESCs (Figure 7A). Meanwhile, the RNA levels of β-Catenin and GSK3β remained unchanged in IHESCs upon IFNA treatment (Figure 7B,C). Compared to the control, NMI knockdown (KD) significantly increased β-Catenin protein levels in immortalized human endometrial stromal cells (IHESCs) and IFNA treatment further elevated β-Catenin protein levels in NMI KD-IHESCs without affecting its RNA expression (Figure 7A,D). Conversely, NMI KD led to a decrease in GSK3β protein levels in IHESCs following IFNA treatment, without a corresponding reduction in its RNA expression (Figure 7A,C). Subsequently, we assessed the inhibition of GSK3β activity through the phosphorylation of serine 9 by Western blot analysis. IFNA treatment alone did not alter the phosphorylated GSK3β (p-GSK3β) to GSK3β ratio in IHESCs (Figure 7A,E). However, in the context of NMI KD and IFNA treatment, there was a significant increase in the p-GSK3β/GSK3β ratio, indicating a reduction in GSK3β activity in IHESCs (Figure 7A,E). Further investigation into how NMI KD affects the expression of β-Catenin target genes revealed that RNA sequencing (RNA-seq) analysis demonstrated a significant upregulation of CCND1 and MMP15 RNA levels in IHESCs with NMI KD compared to the control (Figure 7F). Both CCND1 and MMP15 have an essential role in cell proliferation [47,48]. Therefore, NMI knockdown enhanced the β-catenin/GSK3β axis in endometrial stromal cells, amplifying the effects of IFNA-induced non-canonical pathways, which in turn increased the proliferation of endometriotic lesions.

Subsequently, we explored the effects of NMI knockdown on the IFNA-induced non-canonical PI3K/AKT pathways. In IHESCs, IFNA treatment resulted in an increase in PI3K and AKT protein levels without a corresponding increase in their mRNA levels (Figure 7G,J,K). NMI KD increased the PI3K protein levels, which did not lead to an increase in its mRNA levels (Figure 7G,J). IFNA treatment also increased the levels of phosphorylated PI3K (p-PI3K) in IHESCs (Figure 7G,H). However, NMI KD did not further increase activated PI3K induced by IFNA (Figure 7G,H). In addition, NMI KD did not affect the increase in AKT protein levels induced by IFNA treatment, which also did not lead to an increase in mRNA levels in IHESCs (Figure 7G,K). Despite the elevation of AKT protein levels in IHESCs, IFNA treatment alone did not activate AKT, as indicated by unchanged levels of phosphorylated AKT (p-AKT) at Ser473 (Figure 7G,I). However, NMI KD resulted in an increase in p-AKT levels in IHESCs following IFNA stimulation (Figure 7I). Consequently, NMI KD contributed to the activation of PI3K/AKT signaling in IHESCs, as evidenced by a significant increase in the ratio of p-AKT (Ser473) to AKT in NMI KD-IHESCs upon IFNA treatment (Figure 7G,I).

### 2.7. NMI KD Endometrial Stromal Cells Enhanced the Growth of Ectopic Lesions in Mice with Endometriosis

**Figure 8:**
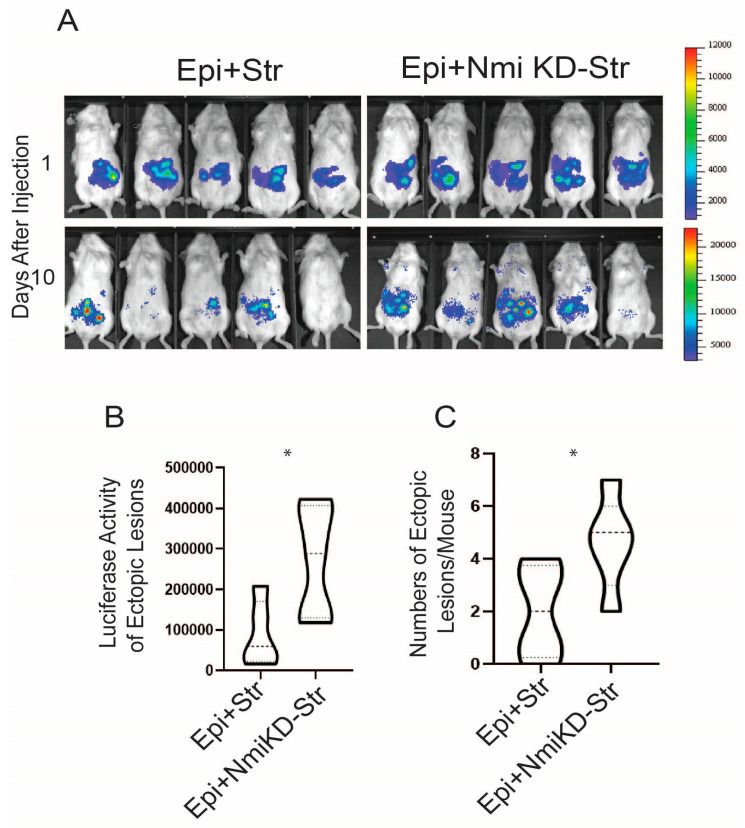
Stimulation of growth of endometriotic lesions in mice by NMI KD endometrial stromal cells. (**A**) Bioluminescence images of endometriotic lesions generated by the mixture of luciferase-labeled immortalized human epithelial cells (LIHEEC) plus luciferase-labeled immortalized human stromal cells (LIHESC) named “Epi+Str” and a mixture of LIHEECS plus NMI KD LIHESCs named Epi+NMI KD-Str in SCID mice at 0 and 10 days post-endometriosis induction. (**B**) Quantification of bioluminescence signals depicted in panel (**A**). (**C**) Quantification of the number of ectopic lesions corresponding to the bioluminescence signals from panel A. Significance levels are indicated as follows: * *p* < 0.05 determined by Students’ *t*-test.

NMI knockdown inhibited IFNA-induced cell death and enhanced non-canonical IFNA pathways in endometrial stromal cells. Consequently, we investigated whether suppressing NMI in these cells could promote the growth of ectopic lesions in NOD-SCID mice, mirroring human endometriosis. To this end, we peritoneally injected mice with a mixture of luciferase-labeled control wild-type endometrial epithelial cells (LIHEECs) and luciferase-labeled control wild-type endometrial stromal cells (LIHECs) (referred to as Epi+Str), along with control wild-type LIHEECs and NMI KD-LIHESCs (referred to as Epi+NMI KD-Str). Since both cell types were luciferase-labeled, we could non-invasively monitor the growth of the injected cell mixtures via luciferase activity. After injection, the mixtures of endometrial stromal and epithelial cells localized well in the pelvic area of the recipient female mice (Figure 8A). On the 10th day of post-endometriosis induction, we assessed the luciferase activity emanating from the human ectopic lesions formed in NOD-SCID mice. This analysis revealed that the mixture containing epithelial cells with NMI KD-stromal cells (Epi+NMI KD-Str) demonstrated a significantly more pronounced development of endometriotic lesions compared to the control mixtures of epithelial and stromal cells, as indicated by the significantly higher luciferase activity in stromal NMI KD endometriotic lesions compared with control lesions (Figure 8A,B). In addition to measuring luciferase activity, we also quantified the number of ectopic lesions per mouse. The combination of epithelial cells with NMI KD-stromal cells (Epi+NMI KD-Str) resulted in a higher count of ectopic lesions than the control mixture of endometrial epithelial and stromal cells (Figure 8A,C). Thus, the suppression of stromal NMI was found to enhance the growth of ectopic lesions in mice models of endometriosis.

## 3. Discussion

In reproductive-aged women, retrograde menstruation leads to the dispersal of endometrial fragments within the pelvic region. Plasmacytoid dendritic cells (pDCs) recognize and present endometrial autoantigens, thereby increasing type I IFN levels [49]. This elevation contributes to the differentiation of monocytes, sustains the survival of dendritic cells, and enhances the activity of cytotoxic T cells [50,51]. These processes potentially enhance the elimination of endometrial fragments in the pelvic area, especially in healthy women [21]. NOD-SCID/γc null mice lack functional T cells, B cells, and NK cells but retain residual populations of human dendritic cells. The reduced dendritic cell activity in NOD-SCID mice is an important factor in utilizing this mouse model for experiments involving human immune system reconstitution [52,53]. Although the exact IFNA levels in these endometriosis mouse models are unknown, IFNA concentrations have been found to range from 100 to 300 pg/mL in the pancreatic lymph nodes of NOD mice [54,55]. Therefore, the NOD-SCID mouse model is the best for determining the effect of IFNA on human endometriosis progression because this mouse produces IFNA through dendritic cells when endometrial cells are injected.

Our findings revealed that exposure to IFNA increased cell death mechanisms, such as apoptosis and necroptosis, in endometrial stromal cells, effectively inhibiting their proliferation. Nonetheless, irregular IFNA signaling is pivotal in the progression of endometriosis, distinguishing affected individuals from healthy women. Notably, the transcription of IFNAR2 is significantly upregulated in the endometrium of women with endometriosis compared to their healthy counterparts [22]. Furthermore, JAK1, a critical component of type I IFN signaling, shows increased expression in endometriosis stromal cells compared to endometrial cells from individuals without endometriosis [23]. A study involving 52 infertile patients diagnosed with moderate or severe endometriosis showed that the intraperitoneal administration of human recombinant IFNA-2b following endometriosis surgery resulted in a recurrence of endometriosis after 21 months [56]. These findings suggest an essential role of type I IFN in endometriosis progression. However, a study presents evidence of inhibiting endometriosis progression by IFNA. For instance, the contested treatment involving human IFNA-2b demonstrated the suppression of proliferation and migration in primary endometrial stromal cells sourced from patients; this treatment attenuates endometriosis in a rat model [57,58].

**Figure 9:**
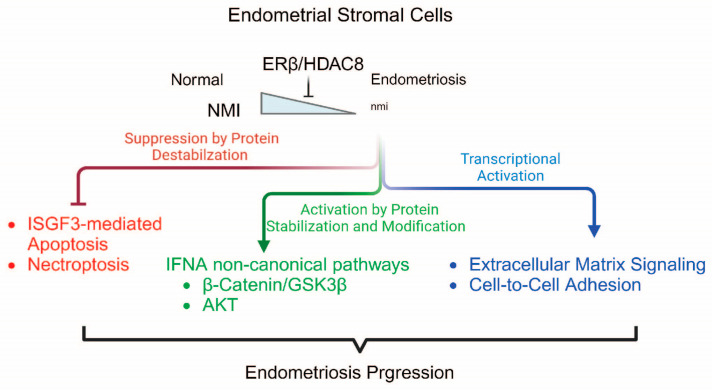
Proposed model of ERβ/HDAC8/NMI axis in endometriosis progression. ERβ/HDAC8 suppresses NMI expression in endometriosis, which subsequently modulates canonical and non-canonical pathways of IFNA.

Our previous study indicated that ERβ inhibits TNFα-induced apoptosis signaling while simultaneously promoting the proliferation, adhesion, and invasion activities of human endometriotic epithelial cells [14]. However, it remains unclear as to how endometriotic stromal cells bypass apoptosis signaling to develop into endometriotic lesions in patients with endometriosis. In this context, we propose that downregulated NMI plays a critical role in the survival of endometrial stromal cells against host immunosurveillance. The downregulation of NMI facilitates the avoidance of IFNA-induced cell death signaling and amplifies IFNA non-canonical pathways within endometrial stromal cells, thereby furthering the progression of endometriosis (Figure 9). Additionally, apart from endometriosis, NMI also exhibits cancer-suppressive properties in various cancers. For example, NMI facilitates the upregulation of dickkopf 1 (Dkk1), an inhibitor of the Wnt/β-catenin signaling pathway. This regulatory action suppresses Wnt/β-catenin signaling and subsequently limits the growth of breast tumors in vivo [27]. NMI also restricts the growth and migration of lung cancer cells by enhancing cell apoptosis [28]. Therefore, NMI exhibits suppressive activity against breast and lung cancer, as well as endometriosis, through a similar mechanism. However, it should be noted that NMI demonstrates an oncogenic function in other types of cancer, such as glioblastoma [59,60]. Hence, NMI possesses a tissue-specific function, dynamically modulating cellular pathways.

IHC analysis of human endometriotic lesions revealed a significant reduction in NMI levels within stromal cells. However, no significant decrease was observed in epithelial cells of ectopic lesions compared to healthy women’s normal endometrium. How does NMI lead to stromal-specific reduction in endometriotic lesions? Understanding the role of down-regulated stromal NMI in endometriosis progression is critical. Histone deacetylation typically silences genes, as HDACs function as transcriptional repressors [61]. HDACs play a significant role in endometriosis. For instance, HDAC inhibition resulted in the reactivation of E-cadherin, attenuation of invasion, and decreased proliferation of endometriotic cells [62]. Trichostatin A, an HDAC inhibitor, suppressed endometriotic lesion growth and hyperalgesia in a mouse model of endometriosis [63]. A recent study revealed that HDAC8 is progressively and aberrantly overexpressed as endometriotic lesions in advance [33,64]. However, this study did not elucidate the role of HDAC8 in the progression of endometriosis. Here, we propose that the ERβ/HDAC8 axis is the key driver for the downregulation of endometrial stromal NMI to enhance endometriosis. Additionally, there is a potent HDAC8-specific inhibitor, PCI-34051, with over 200-fold selectivity over other HDAC isoforms [65]; the treatment with PCI-34051 potentiates antitumor immunity [66]. Our previous study revealed that oleuropein selectively suppressed ERβ compared to ERα in endometriotic lesions [67]. Therefore, the combination of PCI-34051 and oleuropein might effectively suppress the ERβ/HDAC8 axis and consequently restore NMI expression in endometrial stromal cells, rendering them sensitive to IFNA-induced cell death signaling for endometriosis treatment. We will investigate whether the combination of PCI-34051 and oleuropein effectively suppresses the progression of endometriosis.

NMI interacts with various transcription factors and co-regulators to dynamically regulate gene expression [59]. Therefore, the dysregulation of NMI-mediated gene regulation is associated with human disease progression. For example, down-regulation of NMI expression in breast cancer and lung cancers leads to aberrant expression of oncogenes or dysregulation of tumor suppressor genes, contributing to tumorigenesis and cancer progression [28,60]. Therefore, dysregulation of NMI-mediated transcriptional inhibition can enhance oncogenic cellular pathways in these cancers. In the case of endometriosis, NMI effectively suppresses cell-to-cell adhesion and extracellular matrix signaling to prevent endometriosis progression. Thus, dysregulation of NMI-mediated transcriptional inhibition elevates cell-to-cell adhesion and extracellular matrix signaling to enhance endometriosis progression. However, how NMI inhibits the transcription of these genes needs to be addressed. A recent study showed that the interaction of NMI with IFP35 inhibits IFP35 protein degradation [68]. Therefore, NMI directly stabilizes the IFP35 protein through its interaction with IFP35. NMI is known to interact with components of the IRF9/STAT1/STAT2 complex. Consequently, NMI’s integration into the ISGF3 complex components might stabilize these proteins, enhancing ISGF3 complex-mediated IFNA-induced apoptosis. However, how NMI increases protein stability through interaction has not been elucidated yet. In addition to protein stabilization, NMI is also involved in protein destabilization. For instance, NMI interacts with IRF3 and IRF7, promoting the autophagy-mediated degradation of these two transcription factors [68]. In endometriosis, NMI also destabilizes β-catenin in normal endometrial stromal cells. The inhibition of GSK3β stabilized β-catenin protein [69]. Therefore, the stabilization of β-catenin by NMI knockdown would be generated by inactivating GSK3β through an increase in p-GSK3β (Ser9).

NMI was highly expressed in normal lung cells but its expression in lung cancer cells was significantly reduced [28]. This study revealed that overexpression of NMI suppressed lung cancer cell growth and migration by down-regulating phosphorylated PI3K/AKT without changing the protein levels of PI3K and AKT. Conversely, knockdown of NMI promoted lung cancer cell colony formation and migration by increasing phosphorylated PI3K/AKT without reducing their protein levels. In the case of endometriosis, NMI also suppressed PI3K/AKT signaling in normal endometrium to inhibit their growth upon IFNA exposure. Therefore, the downregulation of NMI activates AKT signaling, enhancing endometriosis progression.

In summary, we propose that endometrial stromal NMI potentially acts as a suppressor of endometriosis. Therefore, the loss of endometrial stromal NMI expression, driven by the ERβ/HDAC8 axis, initiates the progression of endometriosis.

## 4. Materials and Methods

### 4.1. Mice

NOD-SCID (NOD.Cg-Prkdcscid/J, Strain number: 001303) female mice (6 weeks old) were obtained from Jackson Laboratory. All mice were housed in the designated animal care facility at Baylor College of Medicine, adhering to the guidelines set by the Institutional Animal Care and Use Committee (IACUC) for the care and use of laboratory animals. The Assurance number is D16-00475. All animal experiments in this study were conducted under an IACUC-approved protocol.

### 4.2. Reagents

Human recombinant IFNα A protein (Sigma-Aldrich, St. Louis, MO, USA, catalog number: IF007) was diluted in 0.1% BSA and added to the cells for 24 h before further analysis.

### 4.3. Cell Culture

We previously isolated and established luciferase-labeled immortalized human endometriotic epithelial cells (IHEEC) and stromal cells (IHESC) from human ovarian endometrioma [14,67]. The cells were cultured in Dulbecco’s Modified Eagle Medium/F-12 (DMEM/F12) supplemented with 10% FBS and maintained in a humidified atmosphere of 5% CO_2_ and 95% air at 37 °C. The medium was replaced every other day.

### 4.4. Generation of Stable NMI Knockdown (KD) Cell Lines

The GIPZ lentiviral shRNA vector targeting human NMI (Clone ID number: V3LHS 323925) was obtained from Open Biosystems. Lentiviral particles were generated in HEK293T cells via transient transfection using the second generation packaging system, which includes the packaging plasmid psPAX2 and envelope plasmid pMD2.G, in conjunction with Lipofectamine 2000 (ThermoFisher, Waltham, MA, USA, Catalog number: 116680300). The titer of the lentiviral particles was determined using Lenti-X™ GoStix™ Plus (Takara Bio, Shiga, Japan, Catalog number: 631280). As a control, a lentivirus containing the non-targeting GIPZ lentiviral shRNA negative control (NT shRNA, Horizon Discovery, Waterbeach, UK, Catalog number: RHS4348) was used for transduction. Both IHEEC and IHESC were infected with lentiviruses carrying either shNMI or NT shRNA using TransDux MAX™ (System Bioscience, Palo Alto, CA, USA, Catalog number: LV860A-1). Subsequently, IHEECs and IHESCs expressing shNMI were selected with 1 µg/mL puromycin. The NMI protein levels in NMI KD IHEECs and NMI KD IHESCs were assessed by Western blot analysis using an NMI antibody (Abcam, catalog number: 183724) compared to NT shRNA IHEECs and IHESCs, which served as WT control endometrial cells.

### 4.5. HDAC8 and NCOR2 Knockdown in IHESCs

To knockdown HDAC8, IHESCs were transfected at a final concentration of 10 nM with the siHDAC8 duplex or control duplex (Origene, Rockville, MD, USA, Catalog number: SR311006) using Lipofectamine 2000 (ThermoFisher, Waltham, MA, USA, Catalog number: 116680300) according to the manufacturer’s instructions. For NCOR2 knockdown, the siNCOR2 duplex (Origene, Rockville, MD, USA, Catalog number: RC212113) was used. Twenty-four hours post-transfection, the cells were supplemented with fresh DMEM/F12 containing 10% charcoal-stripped FBS. After another 24 h, the total RNA was extracted using an RNeasy Plus Mini Kit (Qiagen, Hilden, Germany, catalog number: 74134). The first strand of cDNA was synthesized from 1 μg of RNA of each sample using a SuperScript II™ RT kit (Invitrogen, Carlsbad, CA, USA, Catalog number: 18064022), according to the manufacturer’s protocol. HDAC8 and NMI expression levels were evaluated using quantitative PCR as described below.

### 4.6. Growth Assay of IHECs NMI KD vs. Control

Cells were seeded in 96 well plates at a density of 0.03 × 106 cells/mL. Fresh medium was replaced with either vehicle or 500 or 1000 units/mL IFNα on the following day. After 24 h of treatment, the cells were treated with premix WST-1 (Takara Bio, Shiga, Japan, Catalog number: MK400) to determine cell viability following the manufacturer’s instructions and incubated at 37 °C for 1 h. Their absorbance at 450 nm was measured at 620 nm by a Fisher Scientific accuSkan FC microplate reader. Cell growth was normalized to the vehicle-treated WT. The experiments were conducted in triplicate.

### 4.7. Terminal Deoxynucleotidyl Transferase Biotin-dUTP Nick End Labeling (TUNEL) Assay

Coverslips 12 mm diameter in 24 well plates were coated with 50 µg/mL rat tail Collagen I (Gibco, Billings, MT, USA, Catalog number: A10483-01) diluted in 0.02 M acetic acid for 1 h at 37 °C. IHESCs and NMI KD IHESCs were seeded on the coverslips. Each group was duplicated. On the next day, 0 or 500 units/mL IFNA was added, followed by an additional 24-h incubation. The cells were then subjected to TUNEL assay, using Click-iT™ Plus TUNEL Assay Kits for In Situ Apoptosis Detection with Alexa Fluor™ 594 dye (Invitrogen, Carlsbad, CA, USA, Catalog number: C10618), as per the manufacturer’s instructions. Nuclei were counterstained with Hoechst 33342 (Sigma-Aldrich, St. Louis, MO, USA, Catalog number: B2883). The stained cells were imaged using a Keyence BZ-X800 microscope. Random 6–12 fields of view were captured and counted for TUNEL positive cell percentage.

### 4.8. Reduction in NMI Levels by ERβ in HeLa Cells

According to the manufacturer’s instructions, HeLa cells were transfected with ERβ-expressing plasmids (Addgene, Watertown, MA, USA, Catalog number: 35562) and an empty expression vector (pcDNA 3.1, referred to as MOCK transfection) using Lipofectamine 2000 (ThermoFisher, Waltham, MA, USA, Catalog number: 116680300). Twenty-four hours post-transfection, the cells were supplemented with phenol red-free DMEM containing 10% charcoal-stripped FBS. After another 24 h, estradiol (10 nM) or a vehicle (ethanol) was added, followed by an additional 24-h incubation. The total RNA from cells and tissues was isolated using an RNeasy Plus Mini Kit (Qiagen, Hilden, Germany, catalog number: 74134). The first strand of cDNA was synthesized from 1 μg of RNA of each sample using a SuperScript II™ RT kit (Invitrogen, Carlsbad, CA, USA, Catalog number: 18064022), according to the manufacturer’s protocol. NMI levels were determined by TaqMan probes for NMI (Invitrogen, Carlsbad, CA, USA, Catalog number: Hs00190768_m1). Relative mRNA expression was determined using the 2^−ΔΔCT^ method of quantitative PCR, normalized to 18S rRNA levels. The experiments were conducted in triplicate.

### 4.9. Development of Human Endometriotic Lesions in NOD-SCID Female Mice

NOD-SCID female mice, aged six weeks, were implanted with an estrogen pellet (0.36 mg of 17-β estradiol for 60-day release, Innovative Research of America) to stimulate the progression of endometriosis. The luciferase activity of endometriotic lesions generated by a mixture of WT endometrial epithelial and WT stromal cells was 88,628 ± 80,472.3. However, the luciferase activity of endometriotic lesions generated by a mixture of WT endometrial epithelial and NMI KD stromal cells was 272,370 ± 13,951.9 (Figure 8). A power calculation with these data revealed that the minimum number of mice needed to attain statistical significance of *p* < 0.05 with an 80% probability is five. Thus, five mice per group were used. Following a week of recovery, equal numbers of luciferase-labeled IHEEC (either NMI KD or WT, 1 × 106 cells) and luciferase-labeled IHESC (either NMI KD or WT, 1 × 106 cells) were mixed. This mixture was combined with a Matrigel^®^ basement membrane matrix (Millipore Sigma, Burlington, MA, USA, Catalog number: CLS356234) at a 1:1 ratio and then intraperitoneally injected into NOD-SCID mice implanted with an estrogen pellet. After inducing endometriosis in these mice, bioluminescence imaging was determined to visualize human ectopic lesions. This imaging was performed using the In Vivo Imaging System (IVIS, Perkin Elmer, IVIS^®^ Lumina X5). The experiments were duplicated.

### 4.10. Quantifying Bioluminescence Data

Mice were anesthetized with a 1.5% isoflurane/air mixture using an Inhalation Anesthesia System (VetEquip, Livermore, CA, USA). Next, d-Luciferin (ThermoFisher, Waltham, MA, USA, catalog number: L2916) was intraperitoneally injected at 40 mg/kg mouse body weight. Ten minutes after the D-luciferin injection, the mice were imaged using an IVIS Imaging System (Xenogen, Alameda, CA, USA) with continuous 1% to 2% isoflurane exposure. Imaging variables were maintained for comparative analysis. Grayscale-reflected and pseudocolorized images reflecting bioluminescence were superimposed and analyzed using Living Image software (Version 4.4, Xenogen, Alameda, CA, USA). A region of interest (ROI) was manually selected over the relevant signal intensity regions. The area of the ROI was kept constant across experiments and the intensity was recorded as the total photon counts per second per cm^2^ within the ROI. The measurements were conducted twice per week.

### 4.11. Quantitative PCR (qPCR)

To study relative gene expression, qPCR was conducted using sequence-specific primers in conjunction with the PowerTrack SYBR Green Master Mix (ThermoFisher, catalog number: A46109). Gene-specific primers were designed using the PrimerQuest Tool (https://www.idtdna.com/pages/tools/primerquest (accessed on 25 July 2024)). Relative mRNA expression was determined using the 2^−ΔΔCT^ method of quantitative PCR, normalized to GAPDH levels (Livak and Schmittgen, 2001). Each result is represented by at least three technical replicates. Primer sequences were described in Table S5. The experiments were performed in triplicate.

### 4.12. RNA Sequencing Analysis

WT control IHESC and NMI KD-IHESCs were cultured in 60 mm dishes. Once the cells reached 70–80% confluency, they were incubated with either vehicle or 1000 units/mL IFNα for 24 h. Each condition was performed in triplicate. The total RNA was extracted from IHESC and NMI KD-IHESCs that were treated with either vehicle or IFNα, using the RNeasy Plus Mini Kit (Qiagen, catalog number: 74134), as per the manufacturer’s instructions. To minimize genomic DNA contamination, the RNase spin column membrane was additionally treated with DNase I (2 U/μL). The quality of the total RNA was assessed using the NanoDrop spectrophotometer, Invitrogen Qubit 2.0 quantitation assay, and Agilent Bioanalyzer. Libraries were prepared using the Illumina TruSeq Stranded mRNA library preparation protocol. Sequence reads were trimmed for adapter sequences and low-quality sequences using Galaxy version 23.1.rc1 [70]. Trimmed sequence reads were then mapped to hg38. Subsequently, read count extraction and normalization were conducted on the Galaxy platform. The data were visualized using a heatmap tool in Galaxy version 23.1.rc1.

### 4.13. Western Blotting

Cells were lysed using a homemade 1× RIPA buffer (150 mM NaCl, 50 mM Tris pH 8.0, 1% NP-40, 0.5% Sodium Deoxycholate, 0.1% SDS), supplemented with 1× phosphatase inhibitor cocktails (Gendepot, Katy, TX, USA, catalog number: P3200-001) and 1× Xpert protease inhibitor cocktails (Gendepot, Katy, TX, USA, catalog number: P3100-001). Samples were incubated on ice for 30 min, sonicated for 2 cycles of 10 s on and 20 s off at 20% power, and then centrifuged for 15 min at 15,000 rpm. Equal amounts of proteins were separated by SDS-PAGE and subsequently transferred onto a 0.2 μm PVDF membrane (Cytiva, Marlborough, MA, USA, catalog number: 10-6000-30). The membranes were blocked with 5% skim milk in TBST for 1 h at room temperature and then incubated with antigen-specific primary antibodies in 2.5% skim milk in TBST overnight at 4 °C. After washing, the membranes were incubated with HRP-tagged secondary antibodies (Abcam, Cambridge, UK, catalog number: ab6721) for 1 h at room temperature. Signals were visualized using the SuperSignal™ West Pico Plus Chemiluminescent substrate (ThermoFisher, Waltham, MA, USA, catalog number: 34580). Antibodies used in this experiment were described in Table S6. The experiments were performed in triplicate.

### 4.14. Formalin-Fixed Paraffin-Embedded (FFPE) for Human Endometriotic Lesions and Normal Endometrium

Ovarian endometriomas were removed from patients with endometriosis during surgical procedures at the Baylor College of Medicine, in accordance with an Institutional Review Board (IRB)-approved human protocol. Normal endometrium was isolated from uteri and removed from patients undergoing hysterectomies due to uterine fibroids, also based on an IRB-approved human protocol at the Baylor College of Medicine. All patients had refrained from exogenous hormonal treatments for at least three months prior to their surgeries.

Both endometriotic lesions and normal endometrial samples were fixed in 10% buffered formalin phosphate for 24 h and then stored in 70% EtOH. The tissues were dehydrated with ethanol and xylene using a tissue processor. Following dehydration, the processed tissues were embedded in paraffin.

### 4.15. Immunohistochemistry of NMI in Human Endometriotic Lesions

Tissues preserved in FFPE were sectioned to a thickness of 7 μm. The sliced tissues on the glass slides were deparaffinized in xylene, rehydrated through a gradient of ethanol, and then subjected to immunostaining. Antigen retrieval was carried out using a citrate-based buffer. An antibody against NMI (Abcam, Cambridge, UK, catalog number: 183724) was employed. Specific antigens were visualized using a DAB substrate kit (Vector, Burlingame, CA, USA, catalog number: SK-4100). The intensity of immunostaining was quantified with QuPath software version 0.4.3. (Bankhead, Loughrey et al., 2017 [71]).

### 4.16. Gene Expression Omnibus (GEO)

NMI expression between normal versus endometriotic lesions in human endometriosis patients (GSE25628) was analyzed using Galaxy version 23.1.rc1 [70].

### 4.17. Statistical Analysis

An independent two-tailed Student’s *t*-test was applied for the two-group comparison. In the case of multiple comparisons, a one-way ANOVA with a post-hoc Tukey test was applied. All statistical analyses were performed using GraphPad Prism version 8.0. A *p*-value of <0.05 was considered statistically significant.

## Data Availability

The datasets used and/or analyzed during the current study are available from the corresponding author upon reasonable request. RNA sequencing data regarding the INFA response of NMI KD versus control WT immortalized human endometrial stromal cells has been deposited in the GEO database (GSE241751).

## Supplementary Materials

The supporting information can be downloaded at https://www.mdpi.com/article/10.3390/ijms25158145/s1.

## Abbreviations

Abbreviation	Meaning
ADAM19	ADAM metallopeptidase domain 19
AKT	AKT Serine/Threonine Kinase
BSA	Bovine serum albumin
CDH10	Cadherin 10
CDH15	Cadherin 15
ChIP	Chromation Immuno Precipitation
COL4A1	Collagen Type IV Alpha 1 Chain
COX-2	Cyclooxygenase 2
Dkk1	Dickkopf 1
DMEM	Dulbecco’s minimum essential medium
E2	Estradiol-17β
ELFN1	Extracellular Leucine Rich Repeat And Fibronectin Type III Domain Contain ing 1
ERβ	Estrogen Receptor beta
FBS	Fetal bovine serum
FFPE	Formalin-Fixed Paraffin-Embedded
GSK3β	Glycogen Synthase Kinase 3 Beta
HAPLN3	Hyaluronan And Proteoglycan Link Protein 3
IFNa	Interferon alpha
IFNAR2	Interferon Alpha And Beta Receptor Subunit 2
IHC	Immunohistochemistry
IHEECs	Immortalized human endometrial epithelial cells
IHEECs	ERΒ immortalized human endometrial epithelial cells overexpressing ERβ
IHESCs	Immortalized human endometrial stromal cells
IRF9	Interferon Regulatory Factor 9
ISGF3	IFN-stimulated gene factor 3
ISRE	IFN-stimulated responsive element
ITGB3	Integrin Subunit Beta 3
IVIS	In Vivo Imaging System
JAK1	Janus Kinase 1
JUP	Junction Plakoglobin
KD	Knockdown
MFAP5	Microfibril Associated Protein 5
MLKL	Mixed lineage kinase domain-like
MMP	Matrix Metallopeptidase
MPZL2	Myelin Protein Zero Like 2
NF-kB	Nuclear Factor Kappa B
NMI	N-Myc and STAT Interactor
NMI KD-IHEECs	NMI knockdown immortalized human endometrial epithelial cells
NMI KD-IHESCs	NMI knockdown immortalized human endometrial stromal cells
NOD-SCID	Nonobese diabetic-severe combined immunodeficiency
NT	Non-target
PCDH1	Protocadherin 1
PCDHA1	Protocadherin Alpha 1
PGE2	Prostaglandin E2
PI3K	Phosphoinositide 3-kinases
PML	Promyelocytic leukemia
p-MLKL	phospho-MLKL
p-RIP	phospho-RIP
p-STAT	phospho-STAT
RIP	Receptor-Interacting Serine/Threonine-Protein
SMAD	SMAD Family Member
SORBS1	Sorbin And SH3 Domain Containing 1
SPARC	Secreted Protein Acidic And Cysteine Rich
SPOCK1	SPARC (Osteonectin), Cwcv And Kazal Like Domains Proteoglycan 1
STAT	Signal Transducer And Activator Of Transcription
TBST	Tris-buffered saline
TGF-b	Transforming Growth Factor Beta 1
VCAN	Versican
WT	Wild Type

## References

[1] Zondervan K.T., Becker C.M., Missmer S.A. (2020). Endometriosis. N. Engl. J. Med..

[2] Saunders P.T.K., Horne A.W. (2021). Endometriosis: Etiology, pathobiology, and therapeutic prospects. Cell.

[3] Lyons S.D., Chew S.S., Thomson A.J., Lenart M., Camaris C., Vancaillie T.G., Abbott J.A. (2006). Clinical and quality-of-life outcomes after fertility-sparing laparoscopic surgery with bowel resection for severe endometriosis. J. Minim. Invasive Gynecol..

[4] Zakhari A., Delpero E., McKeown S., Tomlinson G., Bougie O., Murji A. (2021). Endometriosis recurrence following post-operative hormonal suppression: A systematic review and meta-analysis. Hum. Reprod. Update.

[5] Tosti C., Biscione A., Morgante G., Bifulco G., Luisi S., Petraglia F. (2017). Hormonal therapy for endometriosis: From molecular research to bedside. Eur. J. Obs. Gynecol. Reprod. Biol..

[6] Sourial S., Tempest N., Hapangama D.K. (2014). Theories on the pathogenesis of endometriosis. Int. J. Reprod. Med..

[7] Sampson J.A. (1925). Heterotopic or misplaced endometrial tissue. Am. J. Obstet. Gynecol..

[8] Halme J., Hammond M.G., Hulka J.F., Raj S.G., Talbert L.M. (1984). Retrograde menstruation in healthy women and in patients with endometriosis. Obs. Gynecol..

[9] Bulun S.E., Cheng Y.H., Pavone M.E., Xue Q., Attar E., Trukhacheva E., Tokunaga H., Utsunomiya H., Yin P., Luo X. (2010). Estrogen receptor-beta, estrogen receptor-alpha, and progesterone resistance in endometriosis. Semin. Reprod. Med..

[10] Bulun S.E. (2009). Endometriosis. N. Engl. J. Med..

[11] Monnin N., Fattet A.J., Koscinski I. (2023). Endometriosis: Update of Pathophysiology, (Epi) Genetic and Environmental Involvement. Biomedicines.

[12] Xue Q., Lin Z., Cheng Y.H., Huang C.C., Marsh E., Yin P., Milad M.P., Confino E., Reierstad S., Innes J. (2007). Promoter methylation regulates estrogen receptor 2 in human endometrium and endometriosis. Biol. Reprod..

[13] Monsivais D., Dyson M.T., Yin P., Coon J.S., Navarro A., Feng G., Malpani S.S., Ono M., Ercan C.M., Wei J.J. (2014). vERbeta- and prostaglandin E2-regulated pathways integrate cell proliferation via Ras-like and estrogen-regulated growth inhibitor in endometriosis. Mol. Endocrinol..

[14] Han S.J., Jung S.Y., Wu S.P., Hawkins S.M., Park M.J., Kyo S., Qin J., Lydon J.P., Tsai S.Y., Tsai M.J. (2015). Estrogen Receptor beta Modulates Apoptosis Complexes and the Inflammasome to Drive the Pathogenesis of Endometriosis. Cell.

[15] Han S.J., Lee J.E., Cho Y.J., Park M.J., O’Malley B.W. (2019). Genomic Function of Estrogen Receptor beta in Endometriosis. Endocrinology.

[16] Park Y., Han S.J. (2022). Interferon Signaling in the Endometrium and in Endometriosis. Biomolecules.

[17] Shi W., Yao X., Fu Y., Wang Y. (2022). Interferon-alpha and its effects on cancer cell apoptosis. Oncol. Lett..

[18] Shi W.Y., Cao C., Liu L. (2016). Interferon alpha Induces the Apoptosis of Cervical Cancer HeLa Cells by Activating both the Intrinsic Mitochondrial Pathway and Endoplasmic Reticulum Stress-Induced Pathway. Int. J. Mol. Sci..

[19] Herzer K., Hofmann T.G., Teufel A., Schimanski C.C., Moehler M., Kanzler S., Schulze-Bergkamen H., Galle P.R. (2009). IFN-alpha-induced apoptosis in hepatocellular carcinoma involves promyelocytic leukemia protein and TRAIL independently of p53. Cancer Res..

[20] Mazewski C., Perez R.E., Fish E.N., Platanias L.C. (2020). Type I Interferon (IFN)-Regulated Activation of Canonical and Non-Canonical Signaling Pathways. Front. Immunol..

[21] Pia M.M., Anna L.V., Ulla B.K., Koel C., Baidyanath C. (2012). Virus Infection and Type I Interferon in Endometriosis. Endometriosis.

[22] Kao L.C., Germeyer A., Tulac S., Lobo S., Yang J.P., Taylor R.N., Osteen K., Lessey B.A., Giudice L.C. (2003). Expression profiling of endometrium from women with endometriosis reveals candidate genes for disease-based implantation failure and infertility. Endocrinology.

[23] Matsuzaki S., Canis M., Pouly J.L., Botchorishvili R., Déchelotte P.J., Mage G. (2006). Differential expression of genes in eutopic and ectopic endometrium from patients with ovarian endometriosis. Fertil. Steril..

[24] Lebrun S.J., Shpall R.L., Naumovski L. (1998). Interferon-induced upregulation and cytoplasmic localization of Myc-interacting protein Nmi. J. Interferon Cytokine Res..

[25] Xu X., Chai K., Chen Y., Lin Y., Zhang S., Li X., Qiao W., Tan J. (2018). Interferon activates promoter of Nmi gene via interferon regulator factor-1. Mol. Cell Biochem..

[26] Zhu M., John S., Berg M., Leonard W.J. (1999). Functional association of Nmi with Stat5 and Stat1 in IL-2- and IFNgamma-mediated signaling. Cell.

[27] Fillmore R.A., Mitra A., Xi Y., Ju J., Scammell J., Shevde L.A., Samant R.S. (2009). Nmi (N-Myc interactor) inhibits Wnt/beta-catenin signaling and retards tumor growth. Int. J. Cancer.

[28] Wang J., Zou K., Feng X., Chen M., Li C., Tang R., Xuan Y., Luo M., Chen W., Qiu H. (2017). Downregulation of NMI promotes tumor growth and predicts poor prognosis in human lung adenocarcinomas. Mol. Cancer.

[29] Meng D., Chen Y., Yun D., Zhao Y., Wang J., Xu T., Li X., Wang Y., Yuan L., Sun R. (2015). High expression of N-myc (and STAT) interactor predicts poor prognosis and promotes tumor growth in human glioblastoma. Oncotarget.

[30] Mohankumar K., Li X., Sung N., Cho Y.J., Han S.J., Safe S. (2020). Bis-Indole-Derived Nuclear Receptor 4A1 (NR4A1, Nur77) Ligands as Inhibitors of Endometriosis. Endocrinology.

[31] Klinge C.M., Jernigan S.C., Mattingly K.A., Risinger K.E., Zhang J. (2004). Estrogen response element-dependent regulation of transcriptional activation of estrogen receptors alpha and beta by coactivators and corepressors. J. Mol. Endocrinol..

[32] Duong V., Licznar A., Margueron R., Boulle N., Busson M., Lacroix M., Katzenellenbogen B.S., Cavaillès V., Lazennec G. (2006). ERα and ERβ expression and transcriptional activity are differentially regulated by HDAC inhibitors. Oncogene.

[33] Zheng H., Liu X., Guo S.W. (2023). Aberrant expression of histone deacetylase 8 in endometriosis and its potential as a therapeutic target. Reprod. Med. Biol..

[34] Sugihara K., Kobayashi Y., Suzuki A., Tamura N., Motamedchaboki K., Huang C.T., Akama T.O., Pecotte J., Frost P., Bauer C. (2014). Development of pro-apoptotic peptides as potential therapy for peritoneal endometriosis. Nat. Commun..

[35] Omar S.I., Hamza A.M., Eldabah N., Habiba D.A. (2021). IFN-α and TNF-α serum levels and their association with disease severity in Egyptian children and adults with alopecia areata. Int. J. Dermatol..

[36] Liu Y., Ma J., Yang Y., Liu L., Zhu G., Wang X., Wang S., Guo W., Yue Q., Zhao T. (2020). Impact of Interferon-alpha1b (IFN-α1b) on Antitumor Immune Response: An Interpretation of the Promising Therapeutic Effect of IFN-alpha1b on Melanoma. Med. Sci. Monit. Int. Med. J. Exp. Clin. Res..

[37] Punt C.J., Eggermont A.M. (2001). Adjuvant interferon-alpha for melanoma revisited: News from old and new studies. Ann. Oncol. Off. J. Eur. Soc. Med. Oncol..

[38] Sarhan J., Liu B.C., Muendlein H.I., Weindel C.G., Smirnova I., Tang A.Y., Ilyukha V., Sorokin M., Buzdin A., Fitzgerald K.A. (2019). Constitutive interferon signaling maintains critical threshold of MLKL expression to license necroptosis. Cell Death Differ..

[39] McComb S., Cessford E., Alturki N.A., Joseph J., Shutinoski B., Startek J.B., Gamero A.M., Mossman K.L., Sad S. (2014). Type-I interferon signaling through ISGF3 complex is required for sustained Rip3 activation and necroptosis in macrophages. Proc. Natl. Acad. Sci. USA.

[40] Chawla-Sarkar M., Lindner D.J., Liu Y.F., Williams B.R., Sen G.C., Silverman R.H., Borden E.C. (2003). Apoptosis and interferons: Role of interferon-stimulated genes as mediators of apoptosis. Apoptosis.

[41] Vanden Berghe T., Linkermann A., Jouan-Lanhouet S., Walczak H., Vandenabeele P. (2014). Regulated necrosis: The expanding network of non-apoptotic cell death pathways. Nat. Rev. Mol. Cell Biol..

[42] Li W.X. (2008). Canonical and non-canonical JAK-STAT signaling. Trends Cell Biol..

[43] Marineau A., Khan K.A., Servant M.J. (2020). Roles of GSK-3 and beta-Catenin in Antiviral Innate Immune Sensing of Nucleic Acids. Cells.

[44] Madanes D., Bilotas M.A., Bastón J.I., Singla J.J., Meresman G.F., Barañao R.I., Ricci A.G. (2020). PI3K/AKT pathway is altered in the endometriosis patient’s endometrium and presents differences according to severity stage. Gynecol. Endocrinol. Off. J. Int. Soc. Gynecol. Endocrinol..

[45] Pazhohan A., Amidi F., Akbari-Asbagh F., Seyedrezazadeh E., Farzadi L., Khodarahmin M., Mehdinejadiani S., Sobhani A. (2018). The Wnt/β-catenin signaling in endometriosis, the expression of total and active forms of β-catenin, total and inactive forms of glycogen synthase kinase-3β, WNT7a and DICKKOPF-1. Eur. J. Obs. Gynecol. Reprod. Biol..

[46] Santulli P., Marcellin L., Tosti C., Chouzenoux S., Cerles O., Borghese B., Batteux F., Chapron C. (2015). MAP kinases and the inflammatory signaling cascade as targets for the treatment of endometriosis?. Expert. Opin. Ther. Targets.

[47] Alam S., Zunic A., Venkat S., Feigin M.E., Atanassov B.S. (2022). Regulation of Cyclin D1 Degradation by Ubiquitin-Specific Protease 27X Is Critical for Cancer Cell Proliferation and Tumor Growth. Mol. Cancer Res. MCR.

[48] Majali-Martinez A., Hoch D., Tam-Amersdorfer C., Pollheimer J., Glasner A., Ghaffari-Tabrizi-Wizsy N., Beristain A.G., Hiden U., Dieber-Rotheneder M., Desoye G. (2020). Matrix metalloproteinase 15 plays a pivotal role in human first trimester cytotrophoblast invasion and is not altered by maternal obesity. FASEB J. Off. Publ. Fed. Am. Soc. Exp. Biol..

[49] Suen J.L., Chang Y., Shiu Y.S., Hsu C.Y., Sharma P., Chiu C.C., Chen Y.J., Hour T.C., Tsai E.M. (2019). IL-10 from plasmacytoid dendritic cells promotes angiogenesis in the early stage of endometriosis. J. Pathol..

[50] Ali S., Mann-Nüttel R., Schulze A., Richter L., Alferink J., Scheu S. (2019). Sources of Type I Interferons in Infectious Immunity: Plasmacytoid Dendritic Cells Not Always in the Driver’s Seat. Front. Immunol..

[51] Newby B.N., Brusko T.M., Zou B., Atkinson M.A., Clare-Salzler M., Mathews C.E. (2017). Type 1 Interferons Potentiate Human CD8(+) T-Cell Cytotoxicity Through a STAT4- and Granzyme B-Dependent Pathway. Diabetes.

[52] Ito M., Hiramatsu H., Kobayashi K., Suzue K., Kawahata M., Hioki K., Ueyama Y., Koyanagi Y., Sugamura K., Tsuji K. (2002). NOD/SCID/gamma(c)(null) mouse: An excellent recipient mouse model for engraftment of human cells. Blood.

[53] Spranger S., Frankenberger B., Schendel D.J. (2012). NOD/scid IL-2Rg(null) mice: A preclinical model system to evaluate human dendritic cell-based vaccine strategies in vivo. J. Transl. Med..

[54] Sun F., Yang C.L., Wang F.X., Rong S.J., Luo J.H., Lu W.Y., Yue T.T., Wang C.Y., Liu S.W. (2023). Pancreatic draining lymph nodes (PLNs) serve as a pathogenic hub contributing to the development of type 1 diabetes. Cell Biosci..

[55] Li Q., Xu B., Michie S.A., Rubins K.H., Schreriber R.D., McDevitt H.O. (2008). Interferon-alpha initiates type 1 diabetes in nonobese diabetic mice. Proc. Natl. Acad. Sci. USA.

[56] Acien P., Quereda F., Campos A., Gomez-Torres M.J., Velasco I., Gutierrez M. (2002). Use of intraperitoneal interferon alpha-2b therapy after conservative surgery for endometriosis and postoperative medical treatment with depot gonadotropin-releasing hormone analog: A randomized clinical trial. Fertil. Steril..

[57] Dicitore A., Castiglioni S., Saronni D., Gentilini D., Borghi M.O., Stabile S., Vignali M., Di Blasio A.M., Persani L., Vitale G. (2018). Effects of human recombinant type I IFNs (IFN-alpha2b and IFN-beta1a) on growth and migration of primary endometrial stromal cells from women with deeply infiltrating endometriosis: A preliminary study. Eur. J. Obs. Gynecol. Reprod. Biol..

[58] Ingelmo J.M., Quereda F., Acien P. (1999). Intraperitoneal and subcutaneous treatment of experimental endometriosis with recombinant human interferon-alpha-2b in a murine model. Fertil. Steril..

[59] Pruitt H.C., Devine D.J., Samant R.S. (2016). Roles of N-Myc and STAT interactor in cancer: From initiation to dissemination. Int. J. Cancer.

[60] He T., Qiao Y., Yang Q., Chen J., Chen Y., Chen X., Hao Z., Lin M., Shao Z., Wu P. (2022). NMI: A potential biomarker for tumor prognosis and immunotherapy. Front. Pharmacol..

[61] Chen H.P., Zhao Y.T., Zhao T.C. (2015). Histone deacetylases and mechanisms of regulation of gene expression. Crit. Rev. Oncog..

[62] Wu Y., Starzinski-Powitz A., Guo S.W. (2007). Trichostatin A, a histone deacetylase inhibitor, attenuates invasiveness and reactivates E-cadherin expression in immortalized endometriotic cells. Reprod. Sci..

[63] Lu Y., Nie J., Liu X., Zheng Y., Guo S.W. (2010). Trichostatin A, a histone deacetylase inhibitor, reduces lesion growth and hyperalgesia in experimentally induced endometriosis in mice. Hum. Reprod..

[64] Zheng H., Liu X., Guo S.W. (2023). Corroborating evidence for aberrant expression of histone deacetylase 8 in endometriosis. Reprod. Med. Biol..

[65] Balasubramanian S., Ramos J., Luo W., Sirisawad M., Verner E., Buggy J.J. (2008). A novel histone deacetylase 8 (HDAC8)-specific inhibitor PCI-34051 induces apoptosis in T-cell lymphomas. Leukemia.

[66] Yang W., Feng Y., Zhou J., Cheung O.K., Cao J., Wang J., Tang W., Tu Y., Xu L., Wu F. (2021). A selective HDAC8 inhibitor potentiates antitumor immunity and efficacy of immune checkpoint blockade in hepatocellular carcinoma. Sci. Transl. Med..

[67] Park Y., Cho Y.J., Sung N., Park M.J., Guan X., Gibbons W.E., O’Malley B.W., Han S.J. (2022). Oleuropein suppresses endometriosis progression and improves the fertility of mice with endometriosis. J. Biomed. Sci..

[68] Li L., Chen S.N., Wang K.L., Li N., Pang A.N., Liu L.H., Li B., Hou J., Wang S., Nie P. (2023). Interaction of Nmi and IFP35 Promotes Mutual Protein Stabilization and IRF3 and IRF7 Degradation to Suppress Type I IFN Production in Teleost Fish. J. Immunol..

[69] Wu G., Huang H., Garcia Abreu J., He X. (2009). Inhibition of GSK3 phosphorylation of beta-catenin via phosphorylated PPPSPXS motifs of Wnt coreceptor LRP6. PLoS ONE.

[70] Community T.G. (2022). The Galaxy platform for accessible, reproducible and collaborative biomedical analyses: 2022 update. Nucleic Acids Res..

[71] Bankhead P., Loughrey M.B., Fernández J.A., Dombrowski Y., McArt D.G., Dunne P.D., McQuaid S., Gray R.T., Murray L.J., Coleman H.G. (2017). QuPath: Open source software for digital pathology image analysis. Sci. Rep..

